# Comparison of different decellularization protocols for porcine centrum tendineum diaphragmatis and diaphragmatic muscle – a base for effective recellularization

**DOI:** 10.1186/s13036-025-00602-z

**Published:** 2026-01-07

**Authors:** Bruno F. Gaag, Peter Tang, Oliver Klein, Simon Moosburner, Agnes K. Böhm, Theresa Lohmann, Jonas K. Wieland, Victoria Contes, Yijun Zhou, Eriselda Keshi, Luna Haderer, Eric Metzler, Verena Schöwel-Wolf, Simone Spuler, Jens-Carsten Rückert, Johann Pratschke, Igor M. Sauer, Marco N. Andreas, Karl H. Hillebrandt

**Affiliations:** 1 Department of Surgery, Charité – Universitätsmedizin Berlin, Corporate Member of Freie Universität Berlin and Humboldt-Universität zu Berlin, Experimental Surgery, Augustenburger Platz 1, 13353 Berlin, Germany; 2 Charité – Universitätsmedizin Berlin, Core Facility Imaging Mass Spectrometry, 13353 Berlin, Germany; 3 Berlin Institute of Health at Charité – Universitätsmedizin Berlin, BIH Biomedical Innovation Academy, BIH Charité Clinician Scientist Program, Charitéplatz 1, 10117 Berlin, Germany; 4 MyoPax GmbH, Lindenberger Weg 80, 13125 Berlin, Germany; 5 Max-Delbrück-Centrum für Molekulare Medizin in der Helmholtz-Gemeinschaft, Robert-Rössle-Straße 10, 13125 Berlin-Buch, Germany; 6 Charité – Universitätsmedizin Berlin, Corporate Member of Freie Universität Berlin and Humboldt Universität zu Berlin, Cluster of Excellence Matters of Activity. Image Space Material Funded by the Deutsche Forschungsgemeinschaft (DFG, German Research Foundation) Under Germany’s Excellence Strategy - EXC 2025, Augustenburger Platz 1, 13353 Berlin, Germany

**Keywords:** Tissue engineering, Diaphragm, Bio scaffold, Detergent enzymatic treatment, Proteomics, Decellularization, Porcine

## Abstract

Diaphragmatic dysfunction results from a variety of diseases or post-surgical conditions, leading to impaired lung function and high morbidity and mortality. Current repair materials are limited by poor biocompatibility, functional incompatibility and immune reactions. Tissue engineering via decellularization offers a promising approach by preserving the extracellular matrix while reducing host immune response. However, most studies have focused on rodent models. This study evaluates three decellularization protocols using porcine tissues to increase clinical relevance and optimize diaphragm repair strategies. We compared detergent-enzymatic treatment (DET) adapted from murine diaphragm developed by *Andreas et al.* (P1) and two decellularization protocols for larger mammalian diaphragm tissues published by *Barbon et al.* (P2) for human diaphragm and *Deeken et al.* (P3) for porcine tendinous diaphragm. Decellularized samples were analyzed using histological analysis, SEM, DNA and GAG quantification and proteomic analysis. DNA content was reduced in decellularized tissues significantly between native and decellularized tissues (native: 990.30 ng/mg (IQR = 556.20); P1: 31.92 ng/mg (IQR = 40.38), P2: 32.38 ng/mg (IQR = 20.83), P3: 106.40 ng/mg (IQR = 811.32). Proteomics revealed 4,640 conserved proteins (5.41% classified as matrisomal proteins). The protocols showed a 55.4% concordance of the preserved matrisomal fraction (*n* = 139). The highest protein preservation was achieved by P3, followed by P2 and P1. The P1 and P2 protocols are effective in preserving the extracellular matrix while removing cellular components, with no clear preference. Within our laboratory setting, P3 showed decellularization, but did not reach current decellularization standards. This study advances the preparation for clinical translation of a decellularized porcine diaphragm.

**Supplementary information:**

The online version contains supplementary material available at 10.1186/s13036-025-00602-z.

## Background

Diaphragmatic dysfunction, caused by congenital or acquired defects and various pulmonary and muscle-degenerative diseases, is a serious condition with limited treatment options [1]. As the diaphragm is crucial for breathing and other vital functions, its impairment can cause complications such as herniation, reflux, and pulmonary hypertension [2]. Muscular dystrophies, diaphragm weakness and atrophy, often exacerbated by mechanical ventilation, are major causes of morbidity and reduced quality of life, while lacking effective treatments to preserve diaphragm function [2–11].

Diaphragmatic repair materials must be tailored to the size of the defects or can be sutured on directly, while larger defects require complex repair strategies. Traditional meshes often fail due to poor compatibility, functional incompatibility and immune reactions, resulting in recurrence of diaphragmatic hernia [12–18]. An ideal graft would integrate smoothly, mimic native muscle function, and reduce adhesion to prevent complications [1, 19].

Tissue engineering (TE) focuses on creating bio scaffolds that restore or regenerate impaired tissues by mimicking native structures [20–23]. Decellularization, which retains the extracellular matrix (ECM) while removing immunogenic components (e.g. alpha-Gal epitope and nuclear components), supports regeneration and reduces the need for long-term immunosuppression [15, 24–27].

A recent review by *Boehm et a*l. highlighted the latest advances in diaphragmatic tissue engineering [11]. Most of the reviewed studies employed rodent models due to ethical and practical reasons, with only a few focusing on decellularization of samples of larger animals [28–32]. To enhance clinical relevance, research should explore decellularization techniques for diaphragm tissue from larger animals, such as porcine, as a basis for developing medical devices to repair or augment diaphragm defects in humans.

This paper compares three existing protocols for porcine diaphragmatic decellularization: *Barbon et al.,* which used human diaphragm from cadaveric donors; *Deeken et al.* had used a protocol based on *Cartmell et al.* for the tendinous section of the porcine diaphragm; and a protocol previously established by our group for decellularization of rat diaphragms, adapted for porcine tissue [28, 31–34]. These protocols are compared to outline the advantages and disadvantages of different decellularization strategies regarding their impact on structural integrity, while we systematically evaluate their effects on decellularization efficacy and the retained matrisome profile and glycosaminoglycan (GAG) content, aiming to optimize and standardize diaphragmatic tissue engineering approaches. Additionally, we compare the decellularization process of different tissue components – muscle, tendon, and myotendinous junction – which, to our knowledge, has not been thoroughly investigated in porcine diaphragmatic tissue before. The diaphragmatic tissue-specific composite after decellularization could affect their future usage. This comparison provides a foundation for refining protocols to achieve effective yet gentle decellularization that preserves the tissue’s unique regenerative properties [19, 35, 36].

## Methods

### Animals

With approval by the State Office of Health and Local Affairs (Landesamt für Gesundheit und Soziales, Berlin, Germany; Reg. No. L0243/11), all animal work was performed in accordance with local law and university guidelines. Procurement of the porcine diaphragms was performed on 6 male piglets (H.G.E. Service GmbH Langerwisch, Michendorf, Germany) in an age range from 4 – 6 weeks, weighing between 22 – 25 kg.

### Porcine diaphragm procurement and patch acquisition

In accordance with the 3 R principles in the context of animal testing, we performed the removal of the diaphragm following surgery (6–8 h) as part of a surgical skills course for physicians (“*Laparoscopy for Beginners and Advanced Surgeons*”; Aesculap Academy^®^ Berlin, Germany). Animals were kept *nil per os* 12 h before the procedure. After inducing deep anesthesia by injection of ketamine, fentanyl and dehydrobenzperidol, animals were euthanized with T61 (200 mg Embutramide, 50 mg Mebezonium and 5 mg Tetracaine). The diaphragms were stored at −20 °C until further use.

Six porcine diaphragms were thawed in 37 °C water bath and then washed in phosphate-buffered saline (PBS). The *pars lumbalis* was then removed. Approximately 2.5 × 2.5 cm large patches were prepared from the diaphragms. The patches were either muscle (mus), tendon (tend), or the myotendinous junction (mt). The patches were stored at −20 °C in PBS until decellularization or directly processed as native controls. Patches were equally allocated to 9 subgroups. We prepared 6 diaphragmatic patches for each of the subgroups, plus 6 patches per tissue type retrieved from the diaphragm as a native control group. Following experimental groups and tissue subgroups were created:

- *Andreas et al.* modified (protocol 1, **P1**): muscle, tendon, myotendinous junction
- *Barbon et al.* (protocol 2, **P2**): muscle, tendon, myotendinous junction
- *Deeken et al.* (protocol 3, **P3**): muscle, tendon, myotendinous junction
- *Native:* muscle, tendon, myotendinous junction

### Porcine diaphragm decellularization

Due to the greater thickness of porcine diaphragm compared to rat tissue, the protocol by *Andreas et al.* was adapted and will be referred to as **protocol 1 (P1)** [34]. The SDS step was prolonged to 30 h with one change of the SDS solution after 15 h. Furthermore, the concentration of the DNase-I in the second step was doubled to 120 units/mL. Finally, we added penicillin/streptomycin, as an antibacterial measure, during the final washing step with PBS. The protocol of *Barbon et al.*, further **protocol 2 (P2)**, was performed with an adjustment of the DNase-I concentration (120 Units/mL), due to missing information in the protocol description [32]. The protocol of *Deeken et al.* was followed as originally described and is further referred to as **protocol 3 (P3)** [28, 31].

For P1 sodium dodecyl sulfate (SDS, Carl Roth, Karlsruhe, Germany), DNase-I (Roche, Basel, Switzerland) in Hanks’ Balanced Salt Solution (HBSS, Bio&Sell, Feucht, Germany), Triton® X-100 (Carl Roth, Karlsruhe, Germany), penicillin/streptomycin (Gibco, Thermo Scientific, Waltham, USA) and PBS was used. For P2 we used distilled water as aqua dest., DNase-I (Roche, Basel, Switzerland) in NaCl buffer (Carl Roth, Karlsruhe, Germany), 0.05% trypsin + 0.02% ethylenediaminetetraacetate (EDTA) in PBS (PAN BIOTech, Aidenbach, Germany), ammonia solution (Otto Fischar, Saarbrücken, Germany), SDS (Carl Roth, Karlsruhe, Germany), Tergitol™ (Sigma-Aldrich, St. Louis, USA) and PBS was used. In P3 we used tri-n-butyl phosphate (TnbP) (Merck, Darmstadt, Germany) in Tris-buffered saline (TBS) and 70% ethanol (EtOH) (Carl Roth, Karlsruhe, Germany) for decellularization. Orbital shaking (OS) was performed on an orbital shaking plate (Unimax 2010, Heidolph, Schwabach, Germany). The different detergent-enzymatic treatment (DET) steps and concentrations are described in further detail in Table 1.

**Table 1:** Decellularization protocols

	protocol 1	protocol 2	protocol 3
	*Andreas et al.* **modified**	Barbon et al.	Deeken et al.
1.	0.1% SDS in Aqua dest. (15 h, RT, OS 200 rpm)	Aqua dest. (24 h, 4 °C)	Aqua dest. (RT, washing)
2.	0.1% SDS in Aqua dest. (15 h, RT, OS 200 rpm)	DNase-I in 1 M NaCl 120 Units/mL (3 h, RT)	1% TnbP in TBS (24 h, RT, OS 200 rpm)
3.	DNase-I in HBSS 120 Units/mL (1 h, RT, OS 200 rpm)	0.05% Trypsin + 0.02% EDTA in PBS (1 h, 37° C)	1% TnbP in TBS (24 h, RT, OS 200 rpm)
4.	1% Triton X-100 in Aqua dest. (1 h, RT, OS 200 rpm)	0.5% SDS + 0.8% NH4OH in PBS (48 h, 4°C)	Aqua dest. (24 h, RT, OS 200 rpm)
5.	100 Units/ml Penicillin + 100 µg/mL Streptomycin in PBS (20 h, RT, OS 200 rpm)	0.5% Tergitol™ + 0.8% NH4OH in PBS (24 h, 4°C)	70% EtOH (24 h, RT, OS 200 rpm)
6.		Aqua dest. (48 h, 4°C)	

The different protocols used in this study are visualized [31, 32, 34]. Before decellularization patches were stored at − 20 °C in PBS and thawed at 37 °C. After decellularization the patches were either directly processed for histology and SEM or frozen at − 80 °C to later analyze DNA, glycosaminoglycans and proteomics

After decellularization, the patches were divided for analysis and were either directly processed or homogenized and frozen at −80 °C. Frozen samples were later lyophilized.

Samples were used for either a) proteomic analysis, deoxyribonucleic acid (DNA) quantification, GAG quantification (each, *n* = 6), or b) histological preparation (*n* = 6). One sample was processed for scanning electron microscopy (SEM). Native controls were processed in the same manner.

### Histological evaluation

Immediately after decellularization, the samples were fixed in 4% formaldehyde (SAV Liquid Production, Flintsbach am Inn, Germany) for 18 - 24 hours. Dehydration was achieved through automatic rinsing in an ascending alcohol series, followed by embedding the samples in paraffin. For histological analysis, the specimens were cut into 3 µm thin slices and fixed onto Superfrost^®^ object slides (Epredia, Breda, The Netherlands) at 60 °C for 30 minutes.

Histological characterization of the decellularized extracellular matrix (dECM) composites and microscopic validation were performed with hematoxylin and eosin (H.E., AppliChem, Darmstadt, Germany), as well as Elastica-Van-Gieson, Masson’s Trichrome, Sirius Red, and Alcian Blue staining (all from Morphisto, Offenbach, Germany). The specimens were then permanently mounted with Eukitt (AppliChem, Darmstadt, Germany).

Fluorescence staining was performed with 4′,6-diamidino-2-phenylindole (DAPI) (Sigma-Aldrich, St. Louis, USA; Art.-Nr. D9542; 1:500) and mounted with fluorescence mounting medium (DAKO, Agilent Technologies Company, Santa Clara, USA).

For immunohistological staining, antigen retrieval was performed with heated 0.01 M citrate buffer pH 6.0 (Carl Roth, Karlsruhe, Germany), followed by a peroxidase block with 2% H_2_O_2_ in methanol (VWR, Radnor, USA; Carl Roth, Karlsruhe, Germany). A serum-free protein block (DAKO, Agilent Technologies Company, Santa Clara, USA) was performed. As primary antibodies, those listed in Supplementary file 1 were used.

Subsequently, the indirect method using goat anti-rabbit IgG H&L (HRP) (abcam, Cambridge, UK; Art.-Nr. ab6721) as a secondary antibody was used for desmin, collagen IV, fibronectin and laminin.

For elastin and collagen I stain, the LASB^®^ method (DAKO, Agilent Technologies Company, Santa Clara, USA) was used, according to the manufacturer’s instructions. A DAB detection kit (DAKO, Agilent Technologies Company, Santa Clara, USA) was used for detection and prepared according to the manufacturer’s instructions. All sections were counterstained with Mayer’s hematoxylin (AppliChem, Darmstadt, Germany) and permanently mounted with Eukitt (AppliChem, Darmstadt, Germany).

Negative controls for the immunohistochemical stainings were prepared in the same manner as described above, without the application of the primary antibodies (data not shown).

Native controls were processed in the same way as decellularized samples. Detailed protocols of all applied staining methods are available in the *S*upplementary files 2–3.

Microscopic images of the stained slices were obtained with a Keyence BZ-X810 (Keyence, Osaka, Japan) microscope. Representative images of the slides were taken.

### DNA quantification

Approximately 10 mg of the sample was used for DNA quantification. The DNA was isolated using the DNeasy^®^ Blood&Tissue-Kit (Qiagen, Hilden, Germany) according to the manufacturer’s instructions. DNA quantification was conducted using the NanoDrop 2000c spectrophotometer (Thermo Scientific, Waltham 02451, USA) and normalized to dry weight.

### Glycosaminoglycan quantification

The GAG content was measured using a method described by *Farndale et al.* and used for decellularized tissue by *Napierala et al.* [37, 38].

400 µL of a papain buffer (AppliChem, Darmstadt, Germany), as a blank, and a standardized concentration row (0–100 μg/mL), using chondroitin sulfate (Carl Roth, Karlsruhe 76231, Germany), were mixed with 400 µL dimethylmethylene blue (DMMB; AppliChem, Darmstadt 64,291, Germany). 5 mg of the lyophilized sample was spiked with 1 ml papain-puffer and incubated for 18 h at 60 °C. The supernatant liquid (400 µL) was then mixed with 400 µL DMMB. Blank, standard concentration, and the samples were incubated at room temperature for 30 min after spiking with DMMB. All samples were analyzed at 525 nm with the NanoDrop 2000c spectrophotometer (Thermo Scientific, Waltham, USA). A linear standard curve (0 -100 μg/mL) was generated, and the results of the samples were calculated. The coefficient of determination was greater than 95%. Finally, the results were normalized to dry weight.

### Scanning electron microscopy

Immediately after sampling, pieces approximately 1 × 1 cm in size were cut from the patches. The samples were then pinned to a cork plate and immersed in 2.5% glutaraldehyde (Serva, Heidelberg, Germany) in 0.1 M sodium cacodylate buffer (Serva, Heidelberg, Germany) for fixation. They were then incubated at room temperature for 30 min and stored in the fixative at 4 °C.

At the *Charité Core Facility for Electron Microscopy* (CFEM, Charité – Universitätsmedizin Berlin), samples were washed with 0.1 M sodium cacodylate buffer and post-fixed with 1% osmium tetroxide (Electron Microscopy Sciences, Hatfield, USA) for 2 h. This was followed by dehydration in increasing ethanol concentrations and finally treatment with hexamethyldisilane (Merck Millipore, Darmstadt, Germany). Samples were subjected to gold-palladium sputtering (Sputter coater MED 020, Balzer, Bingen) and stored in vacuum prior to scanning electron microscopy (GeminiSEM 300, Carl Zeiss, Oberkochen, Germany).

### Proteomic analysis

Approximately 10 mg lyophilized tissue was taken and ground before further processing for proteomics. For the protein extraction, digestion and peptide desalting were performed using the filter-aided sample preparation (FASP) technique [39, 40]. Peptides were extracted into 20 µL of 0.1% trifluoroacetic acid (TFA) (Thermo Fisher Scientific, USA) after incubation for 15 min at room temperature. Four microliters of each peptide extract were then separated using a 2–44% acetonitrile gradient in 0.1% formic acid (VWR, USA) on an analytical C18 column (Thermo Fisher Scientific, USA) and a trap column (Thermo Fisher Scientific, USA) at a flow rate of 400 nL/min over 60 minutes, followed by detection with a timsTOF HT flex mass spectrometer (Bruker Daltonics, Billerica, USA) equipped with a CaptiveSpray nano-electrospray ion source 2.

DIA-PASEF was performed with the following settings: PASEF mode with 10 PASEF MS/MS scans. The capillary voltage was set to 1600 V and the spectral range of m/z from 100 to 1700 with an ion-mobility range (1/K0) from 0.85 to 1.30 Vs/cm^2^. The ramp and accumulation time were set to 100 ms and the duty cycle close to 100% with a total cycle time of 0.95 s.

MS/MS parameters were set as follows: Collision energy was ramped linearly from 59 eV at 1/K0 = 1.6 Vs/cm2 to 20 eV at 1/K0 = 0.6 Vs/cm^2^. Precursors with charge state from 0 to 5 were selected with the target value of 20,000 and an intensity threshold of 2,500. All precursors reaching the target value in arbitrary units were dynamically excluded.

The diaPASEF raw data were processed using the open-source tool DIA-NN [41, 42]. For the library-free search in silico digest library (contains 22,875 proteins, and 22,718 gene entries from UniProt database) deep learning-based spectra, retention times, and ion mobility (IMs) prediction were performed. This library was then used to reanalyze the DIA runs (matched between runs; MBS). The parameters for library analysis were set to: N-terminal methionine excision and carbamidomethylation, peptide lengths ranging from 7 - 30, precursor charges from 1 - 4, precursor m/z from 300 - 1800, and fragment ion m/z from 200 – 1800 MS1 accuracy > 10 ppm and protein p-value < 1% FDR.

The dataset was then annotated using the Matrisome Analyzer provided by Petrov et al. [43]. The associated genes were used for the annotation. The proteoforms in the dataset, leading to the same gene in the UniProt database, were then annotated manually with “°” and “^” [44]. After primary annotation, protein conservation was considered positive if it was expressed in at least three samples within a given subgroup and visualized using RStudio 2024.12.0 + 467 (Posit Software, PBC, Boston, USA)

### Statistical analysis

For statistical analysis and visualization of DNA and GAG values, GraphPad Prism 10.6.1 (GraphPad Software Inc., San Diego, USA) was used, and the data were expressed as median (interquartile range (IQR) = Q3 - Q1).

The data were tested for normal distribution using the Shapiro-Wilk test. If the data showed normal distribution, the unpaired t-test or ordinary one-way ANOVA was performed, otherwise, the Mann-Whitney test or Kruskal-Wallis test for nonparametric distribution was used. Significance was considered at a p-value ≤ 0.05.

## Results

### Patch assessment

**Fig. 1:**
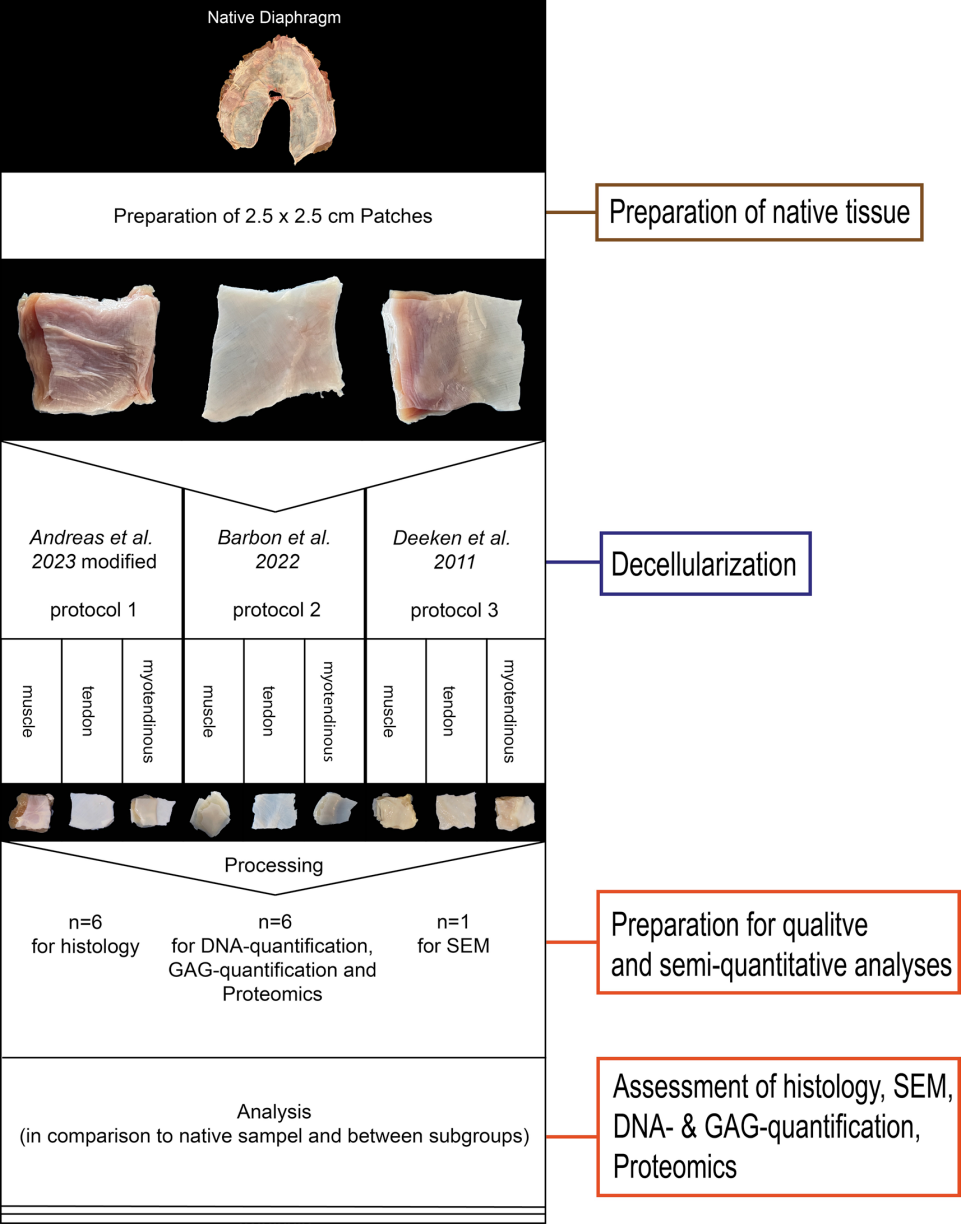
Project workflow. The workflow from preparation of the native tissue, decellularization, processing to analysis is outlined. Before decellularization, the patches were stored in PBS at − 20 °C until use. After decellularization, all patches showed a loss in color. In particular, the tendinous parts appeared to be translucent

**Fig. 2:**
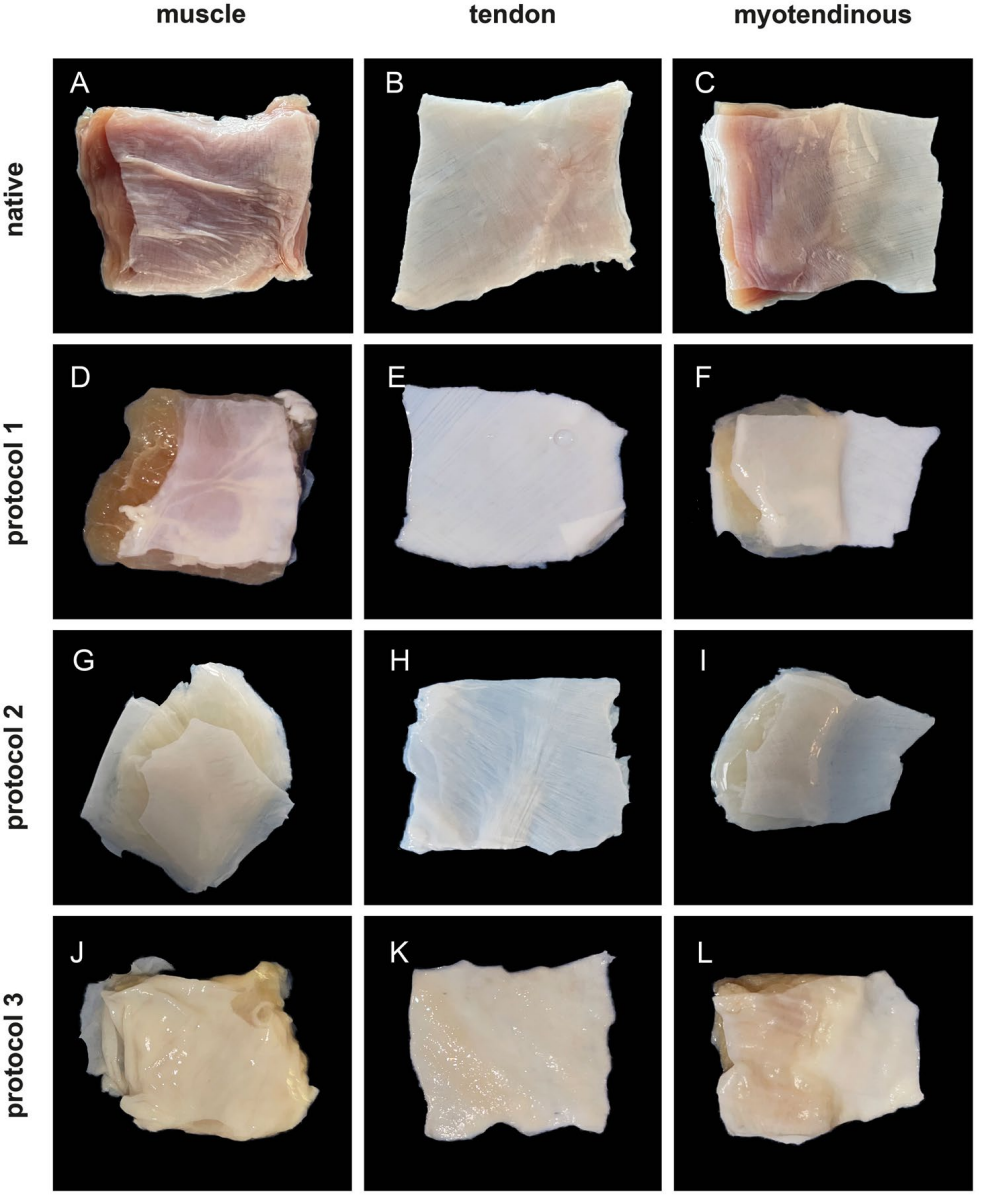
Macroscopic results. Macroscopic images of the native tissue (**A-C**) and after decellularization (**D-L**) are shown. In all groups, the color of the tissue lightened and changed during the decellularization process. Especially the tendinous patches were white after decellularization

The median patch size was 6.30 cm^2^ (IQR = 0.50) while the median length was 2.50 cm (IQR = 0.20), and the median width was 2.50 cm (IQR = 0.10). Native tissue, muscle, tendon, and musculotendinous tissue are shown in Figs. 1 and 2.

The median weight of the patches was 1.40 g (IQR = 1.70). There was no significant difference (*p* = 0.996) in weight between the patches grouped for the native control (1.35 g (IQR = 2.23)), P1 group (1.25 g (IQR = 1.70)), P2 group (1.25 g (IQR = 1.63)) and P3 group (1.60 g (IQR = 1.80)). The median weight of the muscle patches was 2.70 g (IQR = 0.68), in the tendon patches 0.60 g (IQR = 0.30) and in the musculotendinous patches 1.40 g (IQR = 0.48). Again, there were no significant differences between each of the decellularized and native groups (mus: *p* = 0.128; ten: *p* = 0.511; mt: *p* = 0.673). Further details on the weight distribution are displayed in Supplementary file 4.

### Macroscopic evaluation

Macroscopically, all patches showed a color change after decellularization. In the P1 group, the livid red color of the native tissue changed to a lighter red-yellow and was almost translucent in some patches. In the tendon subgroup, all patches were white after decellularization. The muscle patches appeared gelatinous. The patches after decellularization with P2 appeared completely white and were translucent in some parts of the tissue. Compared with other subgroups, the muscle patches seemed to have lost their shape and retained more liquid after decellularization. In the P3 group, the muscle patches appeared more yellow after decellularization, while the tendon parts showed a white color. All patches in the P3 group appeared to be denser and stiffer than those treated with other decellularization methods.

Visualization of the macroscopic decellularization process is illustrated in Fig. 2.

### Microscopic evaluation

#### Histological evaluation

All samples showed a reduction in nuclei compared to the native tissue visible in H.E.-stained samples. P1 group showed no intact nuclei, while the matrix was mostly intact. In muscle portions, the typical fiber structure of skeletal muscle was still visible in most parts of the samples. Similarly, in the tendon parts, the typical structure of tight parallel-fiber connective tissue appeared to be intact.

The samples of the P2 group also showed no intact nuclei. Here, the muscle tissue appeared to be loosened, and in some parts, muscle fibers looked washed out. In the tendinous parts of the samples, the structure of tight parallel-fiber connective tissue appeared to be intact.

In the P3 group, some patches showed no nuclei, while others showed destroyed and reduced intact nuclei. Additionally, a smear of basophilic structure was visible in most of the samples. The overall matrix of muscular and tendinous parts appeared to be most intact in the P3 group, compared with other experimental groups.

DAPI staining revealed a reduction of stainable DNA in all subgroups. The tendinous groups of the P1 and P2 showed no positive responses, while the muscular and combined subgroups showed minor residual DNA between the muscular syncytia, but no intact nuclei, in some samples. In the P3 group, all patches showed a reduced yet positive response to the DAPI staining. In the muscular subgroup, some patches showed positive residual only between the syncytia, while others showed intact nuclei structure. Especially the tendinous subgroup showed residual DNA and nuclei in all parts of the patches. The combined subgroup showed residual DNA in the muscular syncytia and residual DNA and nuclei in the tendinous parts.

The ECM of the tendons and connective tissue appeared to be intact after all decellularization protocols. Regarding the muscle part, P1 and P3 also preserved muscle-specific structures, like the parallel arranged myofibers enclosed by endomysium. However, in samples treated with P2, muscle fibers were reduced, with only the connective tissue remaining.

In the immunohistochemical staining, ECM markers such as collagen I, collagen IV, elastin, and laminin appeared to be preserved in most parts of the samples across all subgroups.

The immunohistochemical staining of the skeletal muscle-specific marker desmin showed no reduction in samples treated with the P3 when compared to native tissue, while the P1 showed a slight reduction of desmin. In the P2 samples, this specific marker was clearly reduced and almost undetectable in areas where the H.E.-staining already indicated a reduction of skeletal muscle fibers.

**Fig. 3:**
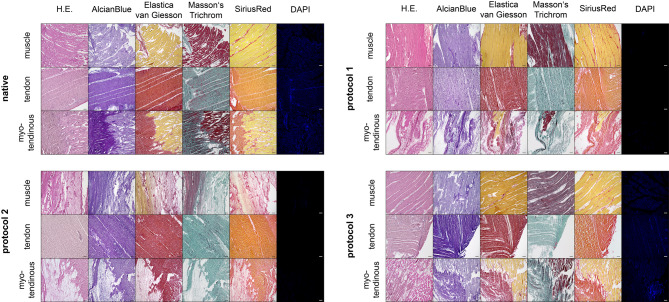
Histology. Histological staining of native and decellularized matrices are shown. Overall, the matrix was best conserved in samples treated with P3. However, P3 showed a positive response in DAPI staining, while the P1 and P2 showed no positive response. In the P1 samples, it appeared that most parts of the matrix were intact, while the P2 samples showed a loosened structure, yet preserving the ECM. Magnification 10x. Scale bar represents 100 µm

**Fig. 4:**
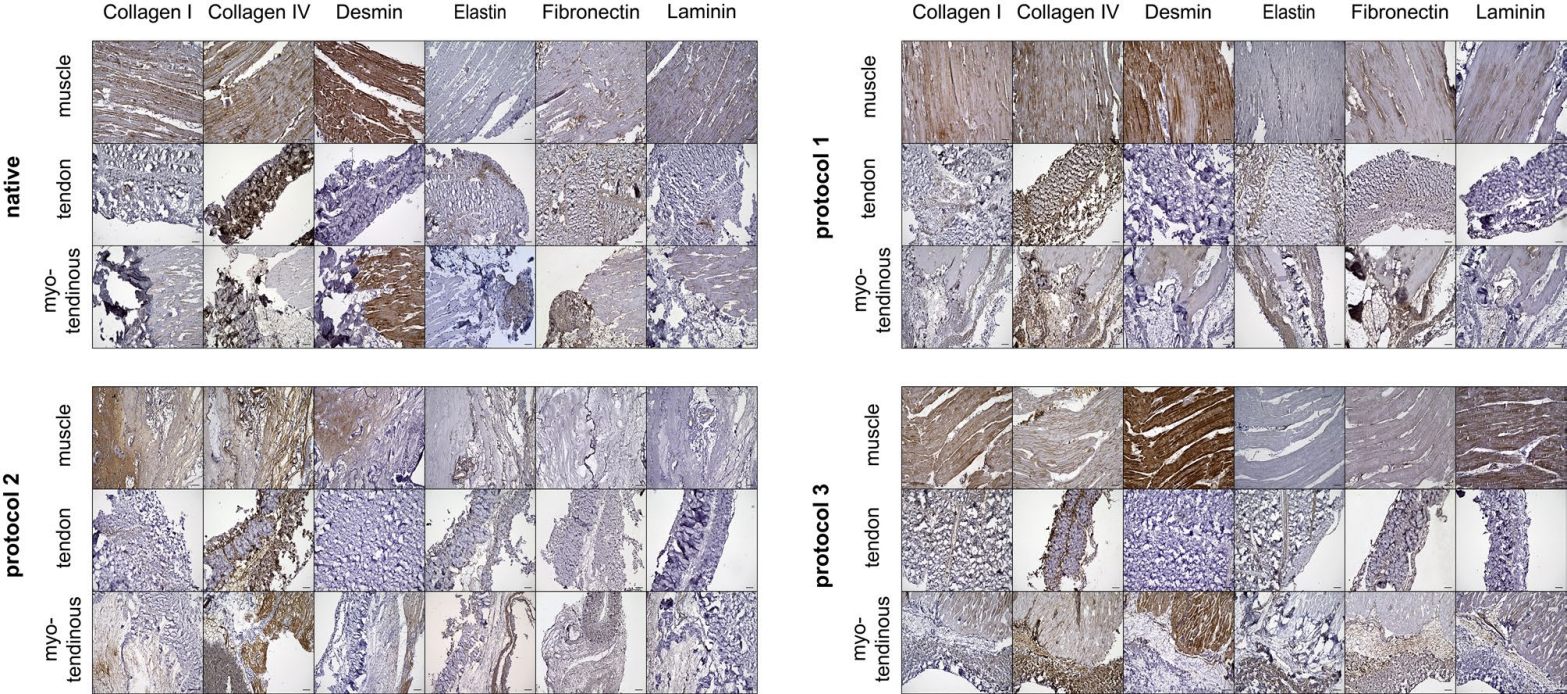
Immunohistochemistry. Immunohistochemistry staining of native and decellularized matrices are shown. Markers of the extracellular matrix, such as collagen I & IV, elastin, fibronectin, or laminin, appear to be preserved to some extent after all decellularization protocols. Muscle-specific markers, like Desmin, were mainly preserved after using P1 and P3, while after P2, they appeared to be reduced. Magnification 10x. Scale bar represents 100 µm

Representative images of all stainings are shown in Figs. 3 and 4.

#### Scanning electron microscopy

**Fig. 5:**
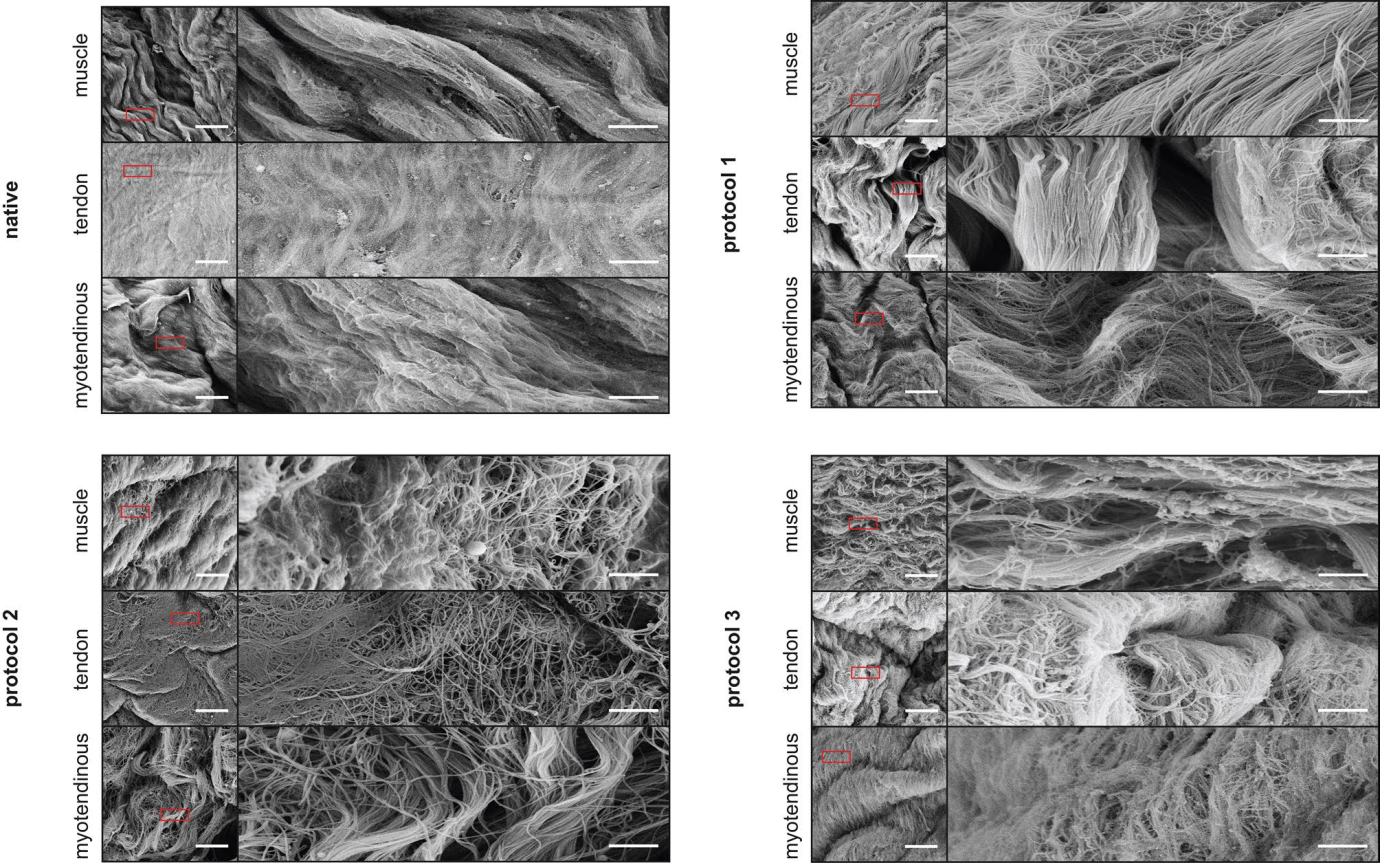
SEM. Exemplary samples from SEM are shown. The overall surface morphology appeared to be intact after decellularization compared to the native samples. Samples from the P1 and P3 groups showed a compact or rather dense structure with some visible disorganized and loosened fibers. After decellularization with P2, samples exhibited a loosened structure with protruding fibrils. Magnification 1,000x (left) and 5,000x (right). Red square indicates the magnification from 1,000x to 5,000x. Figure 4 without marks can be found in Supplementary file 9. Scale bar represents 10 µm (left) and 2 µm (right)

Electron-microscopic analysis (Fig. 5) revealed that the surface morphology remained intact in most regions of all samples. The analysis showed longitudinal rods of collagen fibers arranged in a wavy pattern, along with areas displaying varying degrees of packing density, ranging from looser to denser arrangements.

In the P1 group, both the tendon and myotendinous junction patches showed a compact structure, whereas the muscle patch exhibited visible fibers with disorganized fibrils.

All subgroup patches exhibited a loosened fiber structure, with fibrils protruding in certain areas in the P2 group. The muscular, the tendon, and myotendinous junction patches in the P3 group demonstrated an overall intact fiber structure with loosened fibrils in some regions.

### DNA quantification

DNA quantification was performed with *n* = 6 in each tissue subgroup, totaling *n* = 18 for each experimental group. In the P2 tendon subgroup one sample was excluded due to invalid measurement, resulting in *n* = 5 or rather *n* = 17 for P2 group overall.

All groups showed a decrease in DNA content to varying degrees. The P1 group (31.92 ng/mg (IQR = 40.83); *p* < 0.001) and the P2 group (32.38 ng/mg (IQR = 20.83); *p* < 0.001) achieved a significant decrease in DNA content compared to native samples (990.30 ng/mg(IQR = 556.20)). In the P3 group (106.40 ng/mg (IQR = 811.32); *p* = 0.360) no significant reduction was observed compared to the native samples.

There was no significant difference between the P1 and P2 groups (*p* > 0.999), but a significance to the P3 group (P1 vs. P3: *p* < 0.001; P2 vs. P3: *p* < 0.001).

**Fig. 6:**
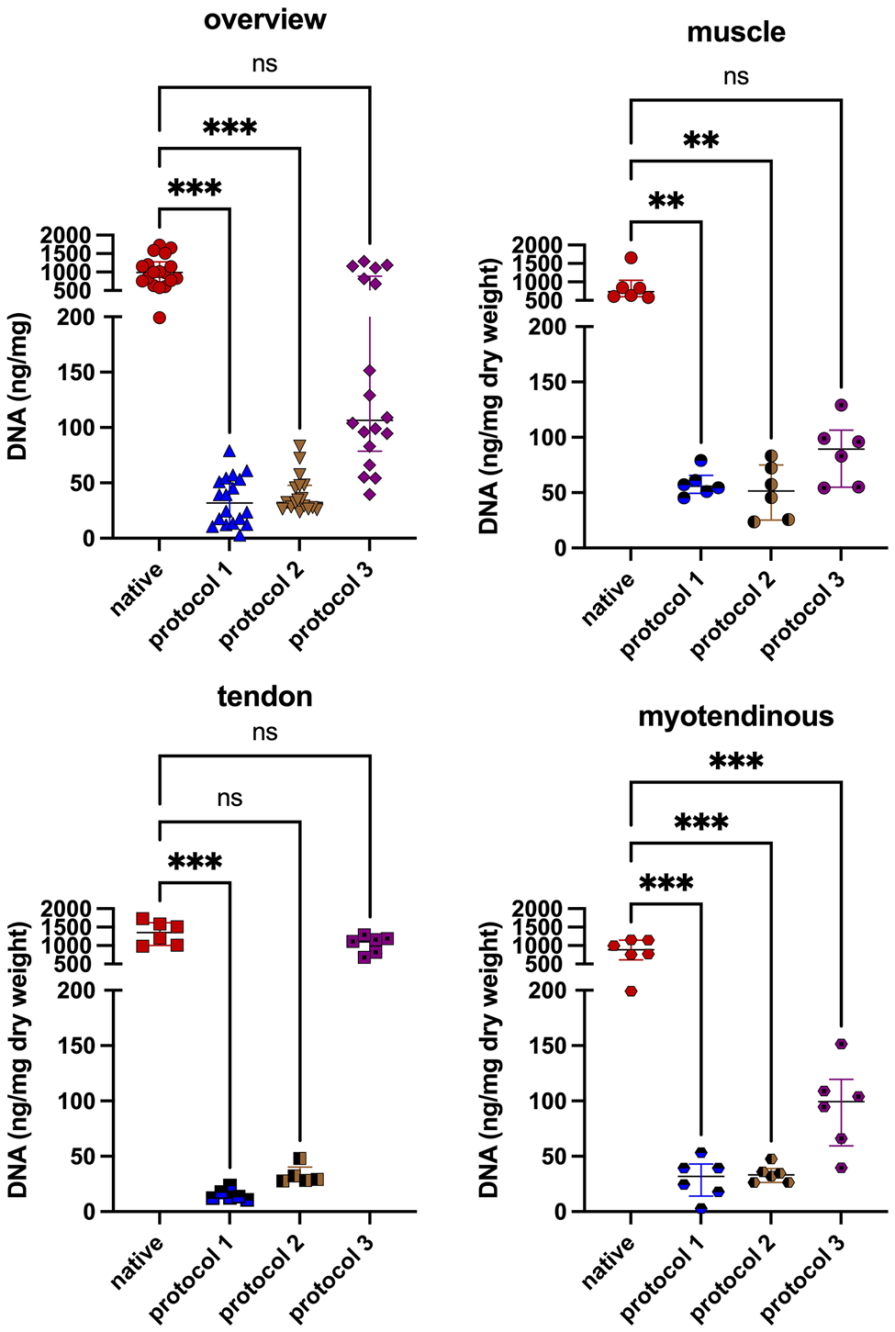
DNA content. After decellularization, samples treated with P1 and P2 showed a significant reduction in DNA. The P3 samples did not reach a point of significant reduction. In the subgroups, the tendinous and combined samples showed lower DNA content than muscle samples in the P1 and P2 groups. In the tendinous group of the P3 samples, almost no reduction of DNA content could be found

A closer look at the tissue subgroups showed that P1 was able to reach a significant reduction in all tissue groups (mus: 55.87 ng/mg (IQR = 16.06) *p* = 0.006; tend: 12.98 ng/mg (IQR = 7.31) *p* < 0.001; mt: 31.92 ng/mg (IQR = 28.88) *p* < 0.001). P2 reached a significant reduction in the muscle and myotendinous group (mus: 51.44 ng/mg (IQR = 49.78) *p* = 0.003; mt: 33.31 ng/mg (IQR = 12.24) *p* < 0.001). However, P2 did not achieve a significance in the tendon group (tend: 29.09 ng/mg (IQR = 12.44) *p* = 0.090). In the P3 groups only the myotendinous group reached a significant reduction (mus: 89.46 ng/mg (IQR = 51.70) *p* = 0.250; tend: 1139 ng/mg (IQR = 429.10) *p* > 0.999; mt: 99.35 ng/mg (IQR = 60.03) *p* < 0.001). The P3 tendon samples exhibited high DNA content and significant difference compared with the muscle (89.46 ng/mg (IQR = 51.70); *p* < 0.001) and myotendinous (99.35 ng/mg (IQR = 60.03); *p* < 0.001) subgroup (Fig. 6).

### Glycosaminoglycan quantification

GAG quantification was conducted with *n* = 6 in each subgroup, adding up to *n* = 18 for each experimental group.

**Fig. 7:**
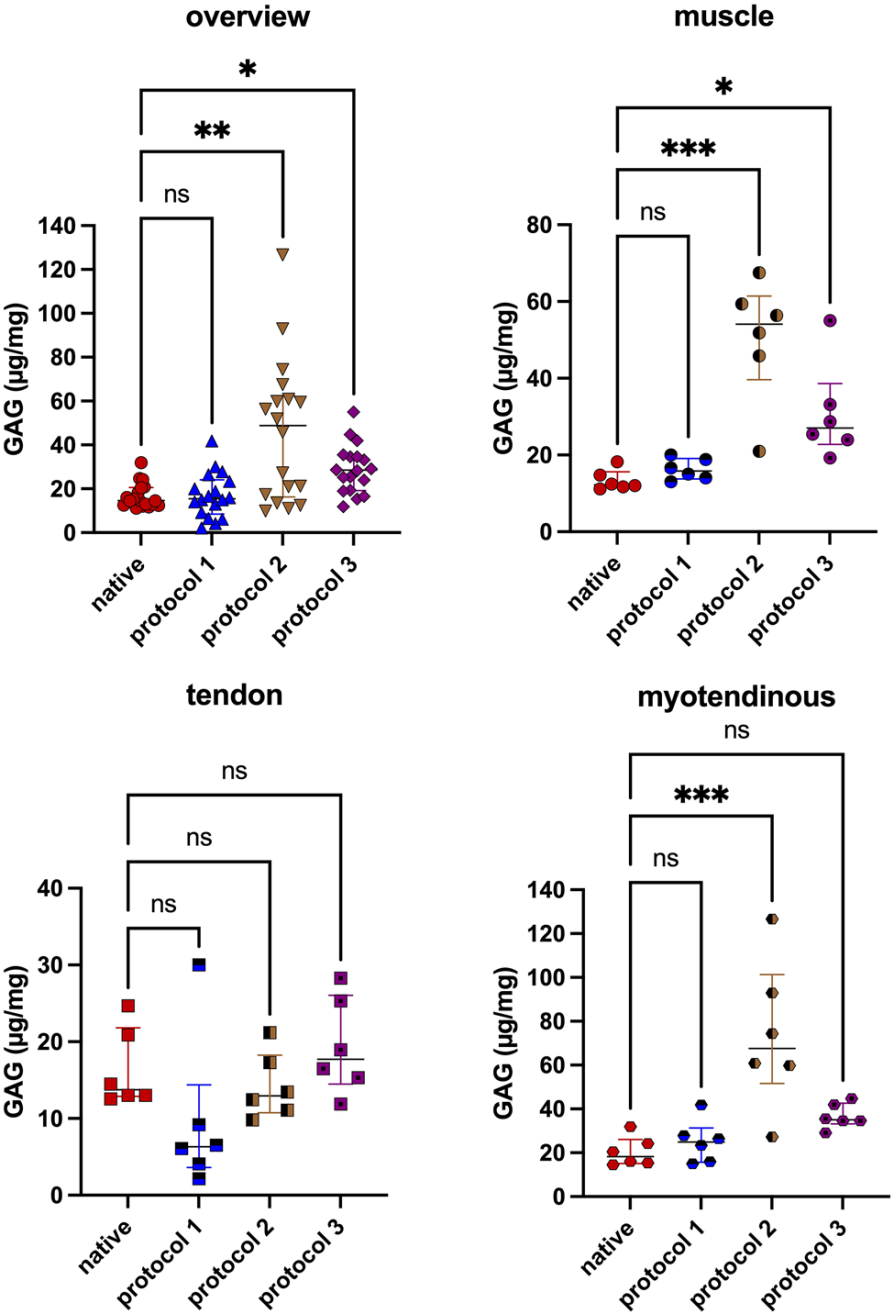
Gag content. Gag content appeared to be stable after decellularization with P1 and higher after treatment with P2 and P3. Looking at the subgroups, the tendinous subgroups appeared to have a lower gag content than the muscular or combined subgroups. These findings were consistent across all decellularization groups

In the P1 group (15.50 µg/mg (IQR = 15.60)) the GAG-content appeared to be stable compared to the native samples (14.66 µg/mg (IQR = 8.07)) and showed no significant difference (*p* > 0.999). The P2 (48.82 µg/mg (IQR = 46.16)) and P3 groups (28.47 µg/mg (IQR = 15.64)) showed higher GAG content compared to the native samples. The difference was significant for both groups (native vs. P2 *p* = 0.004; native vs. P3 *p* = 0.010). Looking at the tissue subgroups, the tendinous samples showed a lower GAG-content in all groups compared to the muscular and myotendinous subgroups after decellularization (Fig. 7).

### Proteomics

The proteome is a complex and heterogeneous mixture of proteins that can be identified in tissue samples and further categorized using a range of analytical tools. The matrisome, as part of the proteome, is divided into two categories: the core matrisome, which includes collagens, ECM glycoproteins, and proteoglycans, and matrisome-associated proteins, which encompass ECM regulatory proteins, ECM-associated proteins, and secreted factors [34, 45].

Following decellularization according to P1, P2 and P3, 4,640 unique proteins were identified. Of these, 139 showed proteoforms leading to the same gene in the UniProt database (raw dataset, dataset with applied rules and proteoforms lists can be found in Supplementary files 6–8) were found. In the dataset, 251 proteins (5.41%) could be matched with MatrisomeDB, provided by *Naba et al.* [45, 46]. Of these, 126 (50.19%) were identified to belong to the core matrisome. Most of them were ECM glycoproteins (*n* = 81; 32.27%), followed by collagens (*n* = 26; 10.36%) and proteoglycans (*n* = 19; 7.57%). Matrisome-associated proteins consisted of ECM regulators (*n* = 62; 24.70%), ECM-affiliated proteins (*n* = 38; 15.14%), and secreted factors (*n* = 25; 9.96%).

All protocols shared a 55.38% concordance in preserved matrisomal fraction (PMF, *n* = 139 proteins), among these, P3 exhibited the highest number of proteins preserved (*n* = 240). P2 and P1 groups exhibited seven and three unique proteins, respectively. However, they shared 14 and 28, respectively, proteins with the P3 group.

Following P1 decellularization, 157 proteins were identified, with 47.77% (*n* = 75) present in all three tissue types. Tendon contained the highest number of PMF proteins (*n* = 127), followed by myotendinous tissue (*n* = 126) and muscles samples (*n* = 101). After P2 decellularization, 175 matrisome proteins were identified. Myotendinous samples had the highest PMF (*n* = 160), followed by muscle (*n* = 148) and tendon (*n* = 95) samples. 164 shared matrisome proteins were identified following P3 decellularization. In this group, tendon had the highest number unique proteins (*n* = 33; 13.75%).

**Fig. 8:**
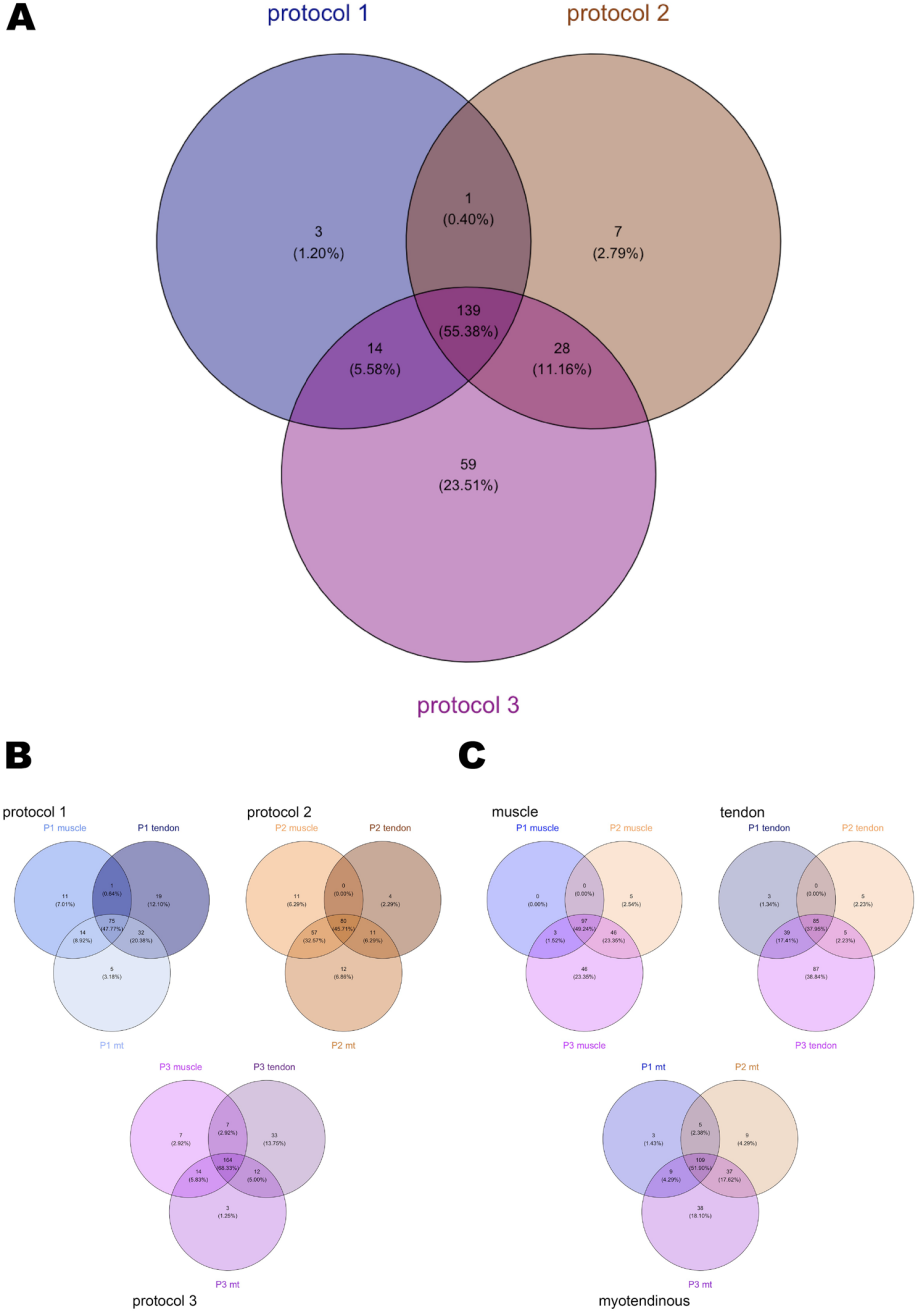
Proteomics I. **A**) number of matrisome proteins shared after decellularization with the three different protocols. Most proteins are shared by all decellularization protocols, while P3 samples had the highest number of uniquely preserved proteins. (**B**) and (**C**) showed the distribution of matrisome proteins in the subgroups. In (**B**) they are grouped by decellularization protocol, in (**C**) by tissue type. In general, the P3 samples showed the highest number of preserved proteins and the largest number of uniquely preserved proteins. A list of matrisome proteins and the subgroups in which they were preserved is shown in the Supplementary file 5. Collagens and ECM glycoproteins were similarly preserved in many subgroups, whereas matrisome-associated proteins showed more diverse pattern of preservation after decellularization

When comparing the tissue subgroups within each protocol group, it was revealed that the muscle subgroups shared 97 matrisome proteins (49.24%), the tendon subgroups shared 85 (37.95%), and the myotendinous junction subgroups shared 109 (51.90%). The P3 subgroups exhibited a higher number of uniquely conserved proteins (mus: *n* = 46, 23.35%; tend: *n* = 87, 38.84%; mt: *n* = 38, 18.10%), whereas the P1 and P2 protocols demonstrated a lower number (*n* = 0–9; 0 - 4.29%) of uniquely conserved proteins (Fig. 8).

Regarding the composition of the matrisomes in the samples, the core matrisome fraction was found to be the most heterogeneous in the P3 tendon group, comprising 69 ECM glycoproteins and 15 proteoglycans. The combined samples from the P2 protocol group had the highest number of collagens, with 23 proteins identified after decellularization. The least heterogeneous mixture of the core matrisome fraction was observed in the P1 muscle samples (14 collagens, 30 ECM glycoproteins, 10 proteoglycans).

**Fig. 9:**
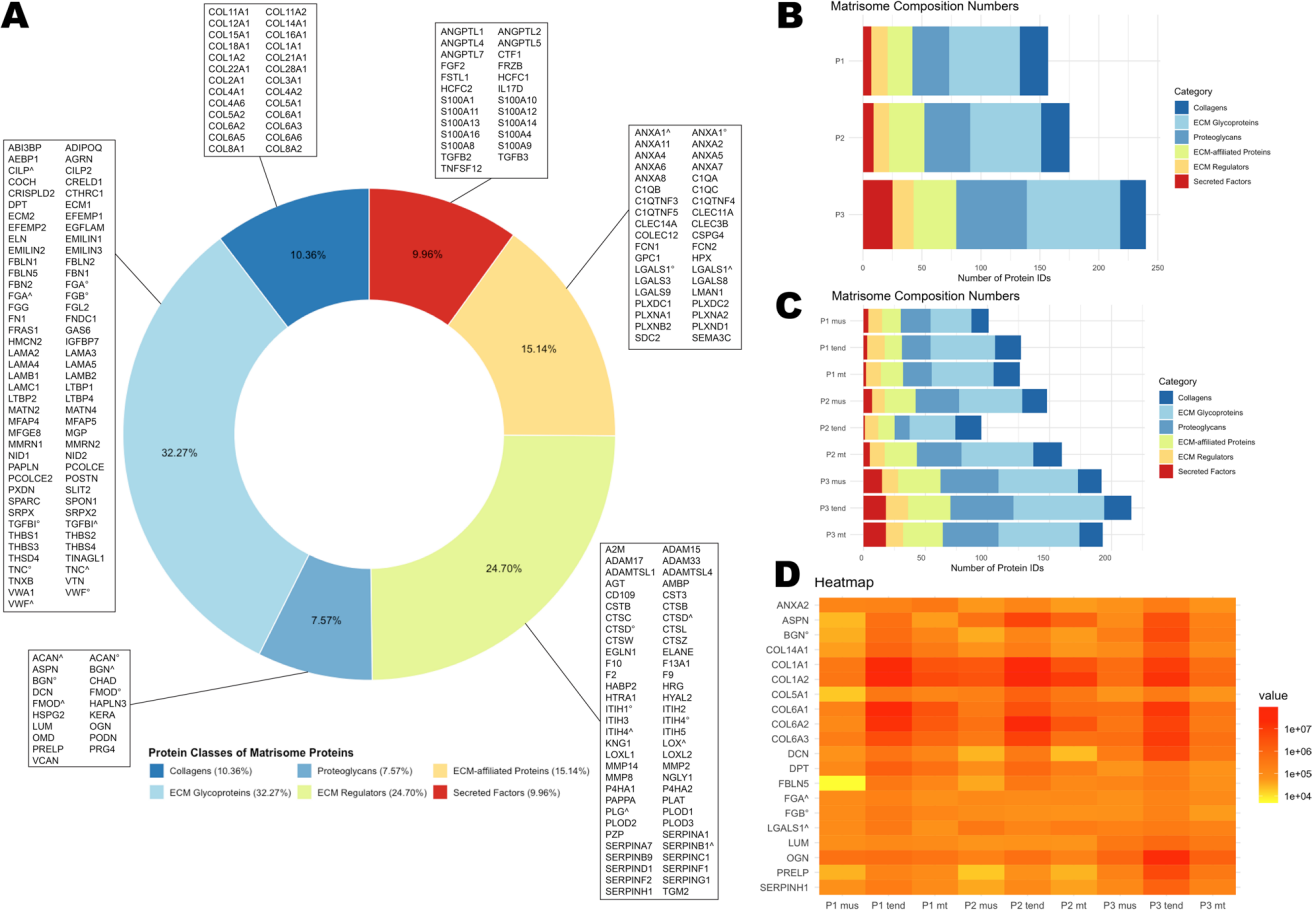
Proteomics II. The distribution of the core matrisome and matrisome-associated proteins of the 251 proteins in the PMF are shown (**A**), annotated with individual protein names. More than 50% of the PMF can be grouped as core matrisome. The individual matrisome distribution is visualized for the decellularization groups (**B**) and subgroups (**C**). the P1 and P2 groups showed a lower number of secreted factors than the P3 samples after decellularization. The collagen fraction and ECM regulators fraction appeared to be mostly stable in all subgroups. In (**D**), the 20 most prevalent matrisome proteins (by median) preserved in all subgroups are demonstrated in a heatmap, showing their median prevalence in each group. Especially COL6A1, COL6A2, COL1A1 and COL1A2 were highly preserved after decellularization. (mus: muscular; tend: tendinous; combi: combined)

P3 subgroups showed the greatest heterogeneity in the matrisome-associated protein fraction, with 51 ECM regulator proteins (P3 tend), 34 ECM-affiliated proteins (P3 tend; P3 mus), and 18 secreted factors (P3 mt; P3 tend). The P2 tendon subgroup had the lowest number of matrisome-associated proteins, with a total of only 12 ECM regulators, 13 ECM-associated proteins, and 1 secreted factor (Fig. 9).

Collagen I (COL1), collagen III (COL3), and collagen VI (COL6) are essential components of the extracellular matrix, each contributing to the structural integrity, elasticity, and functionality of the tissue [47–49]. After decellularization COL1 and COL6 showed preserved content. COL3 also showed preservation in all subgroups. actin (ACTA1), actinin (ACTN2, ACTN3) and myosins (MYH2, MYH3, MYH4, MYL1, MYBPC1, MYBPC2) were preserved to some extent in muscle and combined tissue samples. A specific search for biglycan (BGN), decorin (DCN), and fibromodulin (FMOD) in the dataset showed their presence in all tendon subgroups after decellularization. The tissue-specific protein marker for the myotendinous junction collagen 22A1 (COL22A1) was detected in the P1 and P2 mt samples. Prolargin (PRELP) and periostin (POSTN) were preserved after decellularization in all myotendinous samples.

Semi-quantitative analysis revealed that 61 matrisome proteins were present in all subgroups. 70.49% of these proteins could be classified as core matrisome, comprising primarily collagens and ECM glycoproteins (collagens: *n* = 12, 19.67%; ECM glycoproteins: *n* = 22, 36.06%; proteoglycans: *n* = 9, 14.75%). In the matrisome-associated fraction, only the ECM regulator proteins (*n* = 9, 14.75%) and ECM-affiliated proteins (*n* = 7, 14.75%) were found to be common to all subgroups. The 20 most abundant proteins (by median) shared by all subgroups are presented in a heatmap in Fig. 9.

## Discussion

*Crapo et al.* defined the decellularization criteria as a) the depletion of visible cellular components in H.E. or DAPI, b) *<* 50 ng DNA per mg ECM dry weight, and c) DNA fragment length not exceeding 200 bp [50]. We used the depletion of visible cellular components and a DNA content of *<*50 ng DNA per mg ECM dry weight as an assessment since these two are used by various groups as a standard for consideration of effective matrix decellularization [25, 28, 32, 34, 51]. We observed a significant reduction in DNA content, below the proposed cutoff, in the P1 and P2 treated samples compared to the native samples but could not reach below the threshold in the P3 group. No significant difference was found between the P1 and P2 treated samples. Within subgroups, muscle tissues in P1 and P2 samples had higher DNA content compared to tendon or myotendinous tissues. However, a recent study showed that the endotoxin rather than the residual DNA determines the host response and regeneration behavior of acellular biologic scaffolds [52].

In the P3 group a decrease in DNA was visible, but did not reach significant levels and the determined cutoff. Especially the tendon subgroup had significantly higher DNA content than the muscle and myotendinous samples, treated with the P3 protocol, and did not show DNA reduction within our laboratory setting.

The lower DNA content in tendon and combined samples for P1 and P2 may result from their reduced weight and thickness, potentially enhancing decellularization by altering the sample-detergent relationship. *Reyna et al.* suggested that tissue size and composition strongly influence decellularization, requiring careful analysis for different muscles [53]. Differences in weight, thickness, and tissue type visibly impacted decellularization, supporting the hypothesis that these factors affect the outcome.

Histopathological analyses revealed a reduction in nuclei compared to native tissue in all samples, with varying degrees of structural preservation among the different protocols. It is possible that the remaining cell nuclei and DNA fragments, in the P3 group, are the result of the prolonged ethanol washing step. While ethanol is commonly applied for its antibacterial effect, its dehydrating properties may have a similar effect to formalin, when applied over an extended period, which may have impacted the decellularization efficacy [54]. P1 and P2 utilize PBS or distilled water as the final washing step, which may have benefited the decellularization process. SEM showed that the surface morphology appeared to be intact in most areas of all samples, indicating a structural preservation of the tissue in all three protocols. Typically, the GAG content is expected to be stable or lower after decellularization compared to the native sample [32, 55–58]. We observed a higher GAG content per dry weight after treatment with P2 and P3. This could be an effect of the method, as described by *Napierala et al.* as a possible finding, probably due to a loss of cellular protein mass compared to the native samples [38].

Recent studies have found 368 to 5390 proteins in native porcine muscle or tendon tissue, with a focus on muscle tissue [59–65]. A recent study analyzing ECM hydrogels of the gastrocnemius muscle – Achilles tendon junction was able to detect 2528 proteins in decellularized ECM, with many proteins expressed in both tissue types [66].

We identified a total of 4640 proteins in the decellularized samples combined, of which 251 (5.41%) could be categorized using MatrisomeDB. Decellularization using different protocols resulted in different protein compositions, with P3 preserving the most matrisome proteins. This finding may be related to the residual cellular components observed in DNA- and histological results. Regarding the matrisome preservation in the tissue subtypes, it seemed that protocols favored different tissues. P1 and P3 preserved more matrisome proteins in tendinous samples, while P2 performed better in myotendinous samples. Overall, the combined samples showed only a low number of unique matrisome proteins, indicating that overlapping preservation of the muscle tissue and tendon tissue is represented here.

*Robinson et al.* and *Bi et al.* showed that BGN, DCN and FMOD are important for maintaining tendon homeostasis in terms of collagen fibril structure, fiber realignment and mechanical properties, while BGN and FMOD are important components that organize a cellular niche for tendon stem and progenitor cells [67, 68]. The presence of these proteins after decellularization might provide structural guidance for seeded progenitor cells in an in vitro setting. The myotendinous junction is a specialized unique architectural structure, where the muscle sarcolemma connects to the tendon ECM. While COL22A1 is a marker for myotendinous junctions, PRELP and POSTN are associated with tissue surrounding the junction [69, 70]. The preservation of these proteins in the P1 and P2 myotendinous groups and of PRELP and POSTN in the P3 myotendinous group indicates conservation after decellularization of the extracellular structure typical of the myotendinous junction.

*Boso et al.* showed that their decellularized samples maintained 11% of the matrisome-categorized proteins [47]. 91.82% of their proteins matched our findings in the proteomic analysis. We also observed a highly preserved content of COL1 and COL6 in the matrisome fraction, along with a preservation of COL3 after decellularization. A recent analysis of murine diaphragm, native and decellularized, showed similar results with 1352 proteins and a matrisome fraction of 7.10%, with isoforms of COL1, COL3 and COL6 being the most prevalent in the matrisomal fraction in decellularized tissue [34]. The preserved presence of COL3 may be a supporting factor, and it may provide an optimal regenerative niche for progenitor cells [49]. Preservation of COL6 may also be a beneficial factor upon recellularization, as *Urciuolo et al.* showed that COL6 is a critical component in the satellite cell niche required for preserving self-renewal and muscle regeneration [71]. Some researchers suggest that, in addition to depletion of cell nuclei, the absence of actin and myosin in decellularized muscle tissue indicates sufficient muscle decellularization [1, 53, 58]. Others only use the depletion of nuclei as the major marker for decellularization, while the presence of structural features like the contractile parts are not considered [25, 34]. In the muscular and combined samples actins and myosins were preserved to some extent, while depletion of cell nuclei was achieved in the P1 and P2 samples. The impact of the presence of actin and myosin after decellularization should be investigated in different application settings of decellularized muscle tissue.

While we studied the matrisome fraction of decellularized porcine muscle and tendon tissue, the entire proteomic dataset provides us with a baseline for further analysis in native and decellularized porcine diaphragmatic tissue. The usage of databases, such as STRING database or PANTHER database, to further characterize this tissue-specific proteome should be part of further investigation.

Our study has some limitations. First, we must note that we used the human dataset for matching with MatrisomeDB due to the lack of datasets for porcine samples. This may have resulted in some proteins not being properly matched. However, since the porcine proteome shows a considerable degree of similarity to the human proteome, we accepted this limitation for our analysis [63]. Second, in our proteomic analysis, we considered a result positive only if three or more samples in at least one subset retained the protein. While this serves as a quality marker to avoid false positives in a highly sensitive measurement, it may have led to an underrepresentation of some proteins in our analysis. Third, although we expect the matrix to be suitable for recellularization based on proteomic results and previous experiments with the P2 protocol on human tissue, we did not conduct an in vitro recellularization experiment at this stage of our research [32].

Fourth, in the P2, the concentration of DNase could not be determined. Therefore, we adjusted the concentration to that used in another protocol in our study, which may have affected the reproducibility of the results from the original publication. Fifth, there is a considerable difference between the GAG content of our P2 samples and that of the original publication [32]. We used a different method for processing and analyzing the GAG content, which may have influenced the results and led to these differences.

Sixth, biomechanical testing (BMT) is a valid method for assessing matrix elasticity and is widely used with decellularized tissue. In this study, however, we did not perform BMT. Dissecting the diaphragm into 2.5 × 2.5 cm patches limited the sample geometry for BMT and could have impaired comparability within the groups. Furthermore, the focus of this study was on decellularization efficiency and matrisome preservation, with proteomic characterization, microscopic assessment, and DNA and GAG quantification. However, we would expect that decellularization with P1-3 would have a similar effect on mechanical properties in porcine tissue as in other tissues, since it was tested there [28, 32, 34].

While having some limitations, our experiment’s strength lies in its thorough and systematic comparison of decellularization protocols for different types of porcine diaphragm tissue. It also provides a detailed description of the methods used. Our study further indicates strong evidence that porcine diaphragm-derived scaffolds are well suitable for tissue engineering. We demonstrated that decellularization effectively preserves key extracellular matrix and matrisomal components.

We have found that tendon and muscle samples have unique characteristics after decellularization. They tend to retain tissue-specific differences that were not affected by the use of a specific decellularization method. The differences found using different decellularization protocols may affect the application of the generated matrices. Also, the regenerative effect of the different decellularized matrices may vary after implantation. This study establishes a further foundation for the translational development of a clinically relevant biomaterial. Potential applications include the repair and reconstruction of congenital diaphragmatic hernia and complex soft-tissue defects. Further evaluation in porcine models is crucially needed, as porcine tissue exhibits similarities to human tissue, thereby providing a predictive platform for preclinical testing.

The decellularized scaffolds also provide a foundation for future recellularization approaches using defined cell combinations, supporting functional tissue regeneration. A systematic study of the behavior of muscle progenitor cells on different matrices could prove instrumental in identifying further cellular requirements provided by decellularized matrices.

Standardization of fabrication, mechanical validation, and preclinical implantation studies will be essential for regulatory advancement. Furthermore, the acellular matrices may have the potential to function as off-the-shelf patches, providing a scalable and simple to apply option for surgical reconstruction.

## Conclusion

This work is dedicated to the field of diaphragm tissue engineering. The study involves the review and transfer of established detergent-enzymatic treatment protocols, with the objective of optimize them for porcine tissue. This includes a comprehensive examination of the distinct effects of decellularization on the proteomic composition of tissue types found in porcine diaphragms. The P1 (*Andreas et al., modified*) and P2 (*Barbon et al*.) protocols have demonstrated efficacy in removing cellular components in all diaphragmatic tissue types, while maintaining a distinctive structural and compositional ECM architecture. A comparison of these two protocols suggests that they are equally effective in decellularization. However, it is important to note that the tendon tissue may be more susceptible to adequate decellularization. P3 (*Deeken et al.*) showed decellularization but did not reach current decellularization standards within our laboratory setting. This refinement of protocols, in-depth characterization of decellularized tissues, and disclosure of their matrisomal composition establishes a basis for further investigation into the regenerative potential, due to preserved scaffold structure, and its performance upon recellularization or implantation.

## Electronic supplementary material

Below is the link to the electronic supplementary material.

Supplementary Material 1: Primary antibodies for immunohistochemistry

Supplementary Material 2–3: Protocols of histological and immunohistochemical staining

Supplementary Material 4: Patch assessment

Supplementary Material 5: Heatmap with all samples

Supplementary Material 6: List of matrisome proteins in subgroups

Supplementary Material 7: Proteomic raw data with matrisome annotation

Supplementary Material 8: Proteomic dataset with applied rules & list of isoforms

Supplementary Material 9: Figure 4 without red markings

## Data Availability

The datasets used and analyzed during the current study are available from the corresponding author on reasonable request.

## Abbreviations

ABmod: *Andreas et al.* modified
BMT: Biomechanical testing
COL1: Collagen I
COL3: Collagen III
COL6: Collagen VI
COL22A1: Collagen 22A1
combi: Combined
DAPI: 4′,6-diamidino-2-phenylindole
DB: Database
DET: Detergent-enzymatic treatment
Decm: Decellularized extracellular matrix
DNA: Deoxyribonucleic acid
ECM: Extracellular matrix
EDTA: Ethylenediaminetetraacetate
EtOH: Ethanol
FASP: Filter-aided sample preparation
GAG: Glycosaminoglycan
HBSS: Hanks’ balanced salt solution
H.E.: Hematoxylin-eosin-staining
mus: Muscular
mt: Myotendinous junction
nt: No tendinous samples
OS: Orbital shaking
PBS: Phosphate-buffered saline
PMF: Preserved matrisomal fraction
POSTN: Periostin
PRELP: Prolargin
P1: Protocol 1
P2: Protocol 2
P3: Protocol 3
rpm: Rounds per minute
SDS: Sodium dodecyl sulfate
SEM: Scanning electron microscopy
TBS: Tris-buffered saline
TE: Tissue engineering
tend: Tendinous
TnbP: Tri-n-butyl phosphate
TFA: Trifluoroacetic acid
