# Supplementary material for: Comparison of different decellularization protocols for porcine centrum tendineum diaphragmatis and diaphragmatic muscle – a base for effective recellularization
Source: J Biol Eng. 2026 Jan 7;20:16. doi: 10.1186/s13036-025-00602-z (PMC12836843; doi:10.1186/s13036-025-00602-z)
Supplement: Supplementary file 1 — Supplementary Material 1: Primary antibodies for immunohistochemistry [file 13036_2025_602_MOESM1_ESM.docx]

**Supplementary file 1 - Primary Antibodies for Immunohistochemistry**

| **anti-body** | **manufacturer** | **dilution** |
| --- | --- | --- |
| Rabbit Monoclonal Anti-Desmin (ab32362) | abcam, Cambridge, UK | 1:50 |
| Rabbit Polyclonal Anti-Fibronectin (ab23751) | abcam, Cambridge, UK | 1:150 |
| Rabbit Monoclonal Anti-Laminin (ab11575) | abcam, Cambridge, UK | 1:50 |
| Rabbit Monoclonal, Anti-Collagen IV (ab6586) | abcam, Cambridge, UK | 1:400 |
| Mouse Monoclonal  Anti-Elastin (ab9519) | abcam, Cambridge, UK | 1:100 |
| Mouse Monoclonal Anti-Collagen I (H00001278-M03) | Abnova, Taipei City, Taiwan | 1:400 |
