# Supplementary material for: Comparison of different decellularization protocols for porcine centrum tendineum diaphragmatis and diaphragmatic muscle – a base for effective recellularization
Source: J Biol Eng. 2026 Jan 7;20:16. doi: 10.1186/s13036-025-00602-z (PMC12836843; doi:10.1186/s13036-025-00602-z)
Supplement: Supplementary file 2 — Supplementary Material 2–3: Protocols of histological and immunohistochemical staining [file 13036_2025_602_MOESM2_ESM.docx]

**Supplementary file 2 - Protocols of histological staining.**

| **Name** | **Dewaxing** | **Descending alcohol concentration** | **Staining** | **Differentiation** | **Rinsing** | **Oxidation/**  **Differentiation** | **Staining** | **Rinsing/**  **Differentiation** | **Staining** | **Aldehyde detection** | **Differentiation/**  **Rinsing** | **Staining** | **Rinsing** | **Rinsing** | **Ascending Ethanol concentration** | **Dehyd-ration** | | **Mounting** |
| --- | --- | --- | --- | --- | --- | --- | --- | --- | --- | --- | --- | --- | --- | --- | --- | --- | --- | --- |
| **Alcian Blue- PAS** | Xylol (2x8 min; RT) | 99%, 96%, 90%, 70%, 50%, Aqua dest.   (3 min; RT) | Alcian blue 1% in acetic acid 3%  (5 min; RT) | - | warm Aqua nondest., Aqua dest.  (5 min/ rinsing; RT) | Periodic acid 1%  (10 min; RT) | - | warm Aqua nondest, Aqua dest.  (5 min/ rinsing; RT) | - | Schiff base  (10 min; RT) | warm Aqua nondest, Aqua dest.  (2 min/ rinsing; RT) | Gill-III - Hämatoxylin (1 min; RT) | warm Aqua nondest (1 min; RT) | - | 70%, 96%, 96%, 99%, 99%  (20 sec, 10 sec, 10 sec, 10 sec, 10 sec; RT) | Xylol  (2x2 min; RT) | Eukitt | |
| **Elastica van Gieson** | Xylol (2x8 min; RT) | 99%, 96%, 90%, 70%,  (3 min; RT) | Resorcin-Fuchsin (12 min; RT) | - | Aqua nondest. ( 5 min; RT) | 0,22 % HCL-Alkohol; Aqua nondest. (seconds; 2 min; RT) | Weigert’s iron hematoxylin  (10 min; RT) |  | - | - | 0,22 % HCL-Alkohol; Aqua nondest. (seconds; 8 min; RT) | Van Gieson’s picrofuchsin (1 min; RT) | - | - | 96%, 96%,  99%, 99%  (10 sec, 10 sec, 10 sec, 10 sec; RT) | Xylol  (2x2 min; RT) | Eukitt | |
| **DAPI** | Xylol (2x8 min; RT) | 99%, 96%, 90%, 70%, 50%, Aqua dest.   (3 min; RT) | DAPI  (1:500; 10 min; RT) | - | - | - | - | - | - | - | PBS (5 min; RT) | - | Aqua dest. (RT) | - | - | - | Fluorescence Mounting | |
| **HE** | Xylol (2x8 min; RT) | 99%, 96%, 90%, 70%, 50%, Aqua dest.   (3 min; RT) | Mayer’s hematoxylin  (8 min; RT) | - | warm Aqua nondest. (8 min) |  | - | - | - | - | - | Eosin  (1 min; RT) | PBS (5 min; RT) | Aqua dest. (RT) | 70%, 96%, 96%, 99%, 99%  (5 sec, 5 sec, 10 sec, 20 sec, 20 sec; RT) | Xylol  (2x2 min) | Eukitt | |
| **Mason’s trichrome** | Xylol (2x8 min; RT) | 99%, 96%, 90%, 70%, 50%, Aqua dest.   (3 min; RT) | Weigert’s iron hematoxylin  (15 min; RT) | 0,22 % HCL-Alkohol (seconds; RT) | warm Aqua nondest. (8 min) | - | Acid Fuchsine - Ponceau Azophloxine  (4 min; RT) | Acetic acid 1%  (seconds; RT) | Phophormolyb-den acid – Orange G  (12 min; RT) | - | Acetic acid 1%  (seconds; RT) | Lightgreen - Goldner III (3 min; RT) | Acetic acid 1%  (seconds; RT) | - | 96%, 96%,  99%, 99%  (10 sec, 10 sec, 10 sec, 10 sec; RT) | Xylol  (2x2 min) | Eukitt | |
| **Sirius-Red** | Xylol (2x8 min; RT) | 99%, 96%, 90%, 70%, 50%, Aqua dest.   (3 min; RT) | Weigert’s iron hematoxylin  (10 min; RT) | - | warm Aqua nondest. (5 min) | - | Picro-sirius red  (30 min; RT) | Acetic acid 30%  (1 min; RT) | - | - | - | - | - | - | 96%, 96%,  99%, 99%  (10 sec, 10 sec, 10 sec, 10 sec; RT) | Xylol  (2x2 min) | Eukitt | |

| **Name** | **Dewaxing** | **Descending alcohol concentration** | **Antigen retrieval** | **Rinsing** | **Block** | **Rinsing** | **Block** | **Incubation with primary antibody** | **Rinsing** | **Incubation with secondary antibody** | **Rinsing** | **Detection** | **Rinsing** | **Counter-staining** | **Rinsing** | **Ascending alcohol concentration** | **Dehydration** | **Mounting** |
| --- | --- | --- | --- | --- | --- | --- | --- | --- | --- | --- | --- | --- | --- | --- | --- | --- | --- | --- |
| **Collagen I** | Xylol (8 min, RT) | 99%, 96%, 90%, 70%, 50%, Aqua dest.   (3min; RT) | 0.01 M citrate buffer ph6 (20min; Microwave 360W) | Aqua dest. (2x10min; RT) | 2% H2O2 in Methanol (8min; RT) | PBS  (5min; RT) | Protein Block, Serum-Free  (10min; RT) | Anti-Collagen I (Abnova, H00001278-M03)  in Antibody Diluent (1:400; overnight; 4°C) | PBS  (2x5min; RT) | LASB2®- System-HRP (Dako, K0675) | PBS (5 min; RT) | DAB Detection Kit  (10min; RT) | Aqua dest.  (RT) | Mayer’s Hematoxylin (8min; RT) | warm Aqua nondest. (5 min) | 70%, 96%, 96%, 99%, 99% (45 sec; RT) | Xylol (2x3min; RT) | Eukitt |
| **Collagen IV** | Xylol (8 min, RT) | 99%, 96%, 90%, 70%, 50%, Aqua dest.   (3min; RT) | 0.01 M citrate buffer ph6 (20min; Microwave 360W) | Aqua dest. (2x10min; RT) | 2% H2O2 in Methanol (8min; RT) | PBS  (5min; RT) | Protein Block, Serum-Free  (10min; RT) | Anti-Collagen IV (Abcam, ab6586)  in Anti-Body-Diluent (1:400; overnight; 4°C) | PBS  (2x5min; RT) | Goat Anti-Rabbit IgG H&L (Abcam, ab6721)  in Anti-Body-Diluent (1:400; 45 min; RT) | PBS (5 min; RT) | DAB Detection Kit  (10min; RT) | Aqua dest.  (RT) | Mayer’s Hematoxylin (8min; RT) | warm Aqua nondest. (5 min) | 70%, 96%, 96%, 99%, 99% (45 sec; RT) | Xylol (2x3min; RT) | Eukitt |
| **Desmin** | Xylol (8 min, RT) | 99%, 96%, 90%, 70%, 50%, Aqua dest.   (3min; RT) | 0.01 M citrate buffer ph6 (20min; Microwave 360W) | Aqua dest. (2x10min; RT) | 2% H2O2 in Methanol (8min; RT) | PBS  (5min; RT) | Protein Block, Serum-Free  (10min; RT) | Anti-Desmin (ab32362)  in Anti-Body-Diluent (1:50; overnight; 4°C) | PBS  (2x5min; RT) | Goat Anti-Rabbit IgG H&L (Abcam, ab6721)  in Anti-Body-Diluent (1:400; 45 min; RT) | PBS (5 min; RT) | DAB Detection Kit  (10min; RT) | Aqua dest.  (RT) | Mayer’s Hematoxylin (8min; RT) | warm Aqua nondest. (5 min) | 70%, 96%, 96%, 99%, 99% (45 sec; RT) | Xylol (2x3min; RT) | Eukitt |
| **Elastin** | Xylol (8 min, RT) | 99%, 96%, 90%, 70%, 50%, Aqua dest.   (3min; RT) | 0.01 M citrate buffer ph6 (20min; Microwave 360W) | Aqua dest. (2x10min; RT) | 2% H2O2 in Methanol (8min; RT) | PBS  (5min; RT) | Protein Block, Serum-Free  (10min; RT) | Anti-Elastin (Abcam, ab9519) in Anti-Body-Diluent (1:100; overnight; 4°C) | PBS  (2x5min; RT) | LASB2®- System-HRP (Dako, K0675) | PBS (5 min; RT) | DAB Detection Kit  (10min; RT) | Aqua dest.  (RT) | Mayer’s Hematoxylin (8min; RT) | warm Aqua nondest. (5 min) | 70%, 96%, 96%, 99%, 99% (45 sec; RT) | Xylol (2x3min; RT) | Eukitt |
| **Fibronectin** | Xylol (8 min, RT) | 99%, 96%, 90%, 70%, 50%, Aqua dest.   (3min; RT) | 0.01 M citrate buffer ph6 (20min; Microwave 360W) | Aqua dest. (2x10min; RT) | 2% H2O2 in Methanol (8min; RT) | PBS  (5min; RT) | Protein Block, Serum-Free  (10min; RT) | Anti-Fibronectin (Abcam, ab23751) in Anti-Body-Diluent (1:150; overnight; 4°C) | PBS  (2x5min; RT) | Goat Anti-Rabbit IgG H&L (Abcam, ab6721)  in Anti-Body-Diluent (1:400; 45 min; RT) | PBS (5 min; RT) | DAB Detection Kit  (10min; RT) | Aqua dest.  (RT) | Mayer’s Hematoxylin (8min; RT) | warm Aqua nondest. (5 min) | 70%, 96%, 96%, 99%, 99% (45 sec; RT) | Xylol (2x3min; RT) | Eukitt |
| **Laminin** | Xylol (8 min, RT) | 99%, 96%, 90%, 70%, 50%, Aqua dest.   (3min; RT) | 0.01 M citrate buffer ph6 (20min; Microwave 360W) | Aqua dest. (2x10min; RT) | 2% H2O2 in Methanol (8min; RT) | PBS  (5min; RT) | Protein Block, Serum-Free  (10min; RT) | Anti-Laminin (Abcam, ab11575) in Anti-Body-Diluent (1:50; overnight; 4°C) | PBS  (2x5min; RT) | Goat Anti-Rabbit IgG H&L (Abcam, ab6721)  in Anti-Body-Diluent (1:400; 45 min; RT) | PBS (5 min; RT) | DAB Detection Kit  (10min; RT) | Aqua dest.  (RT) | Mayer’s Hematoxylin (8min; RT) | warm Aqua nondest. (5 min) | 70%, 96%, 96%, 99%, 99% (45 sec; RT) | Xylol (2x3min; RT) | Eukitt |

***Supplementary file 3 - Protocols of immunohistochemical staining.***
