# Supplementary material for: Comparison of different decellularization protocols for porcine centrum tendineum diaphragmatis and diaphragmatic muscle – a base for effective recellularization
Source: J Biol Eng. 2026 Jan 7;20:16. doi: 10.1186/s13036-025-00602-z (PMC12836843; doi:10.1186/s13036-025-00602-z)
Supplement: Supplementary file 3 — Supplementary Material 4: Patch assessment [file 13036_2025_602_MOESM3_ESM.docx]

***Supplementary file 4 – Patch assessment***

| **patch size** |  |
| --- | --- |
| size | 6.30 ±2.20 cm^2^ |
| length | 2.50 ±0.70 cm |
| width | 2.50 ±1.00 cm |
| **weight** |  |
| overall | 1.40 ±3.80 g |
| **Protocol 1** |  |
| overall | 1.25 ±2.70 g |
| muscular | 2.40 ±1.40 g |
| tendinous | 0.60 ±0.30 g |
| musculotendinous | 1.25 ±1.50 g |
| **Protocol 2** |  |
| overall | 1.25 ±2.70 g |
| muscular | 2.35 ±1.30 g |
| tendinous | 0.55 ±0.20 g |
| musculotendinous | 1.25 ±0.50 g |
| **Protocol 3** |  |
| overall | 1.60 ±2.90 g |
| muscular | 2.55 ±1.50 g |
| tendinous | 0.45 ±0.40 g |
| musculotendinous | 1.60 ±1.70 g |
| **native** |  |
| overall | 1.35 ±3.80 g |
| muscular | 3.00 ±1.40 g |
| tendinous | 0.45 ±0.40 g |
| musculotendinous | 1.35 ±0.90 g |

Shown is the median patch size, length, and width. Also, the weight distribution between all subgroups. Size is shown in *cm^2^*, ranges in *cm* and weight in *g*.
