# Supplementary material for: Comparison of different decellularization protocols for porcine centrum tendineum diaphragmatis and diaphragmatic muscle – a base for effective recellularization
Source: J Biol Eng. 2026 Jan 7;20:16. doi: 10.1186/s13036-025-00602-z (PMC12836843; doi:10.1186/s13036-025-00602-z)

**Supplementary file 6 – Heatmap of all Samples**

Shown is the extended heat map from Figure 8 - Proteomics II with the values of all samples represented. Each line represents one sample. The same trend for the distribution as in the simplified heatmap can be seen.

**Protocol 1**

- muscle: 1 – 6

- tendon: 7 – 12

- myotendinous: 13 – 18

**Protocol 2**

- muscle: 19 – 24

- tendon: 25 – 30

- myotendinous: 31 – 36

**Protocol 3**

- muscle: 36 – 42

- tendon: 43 – 48

- myotendinous: 49 – 54


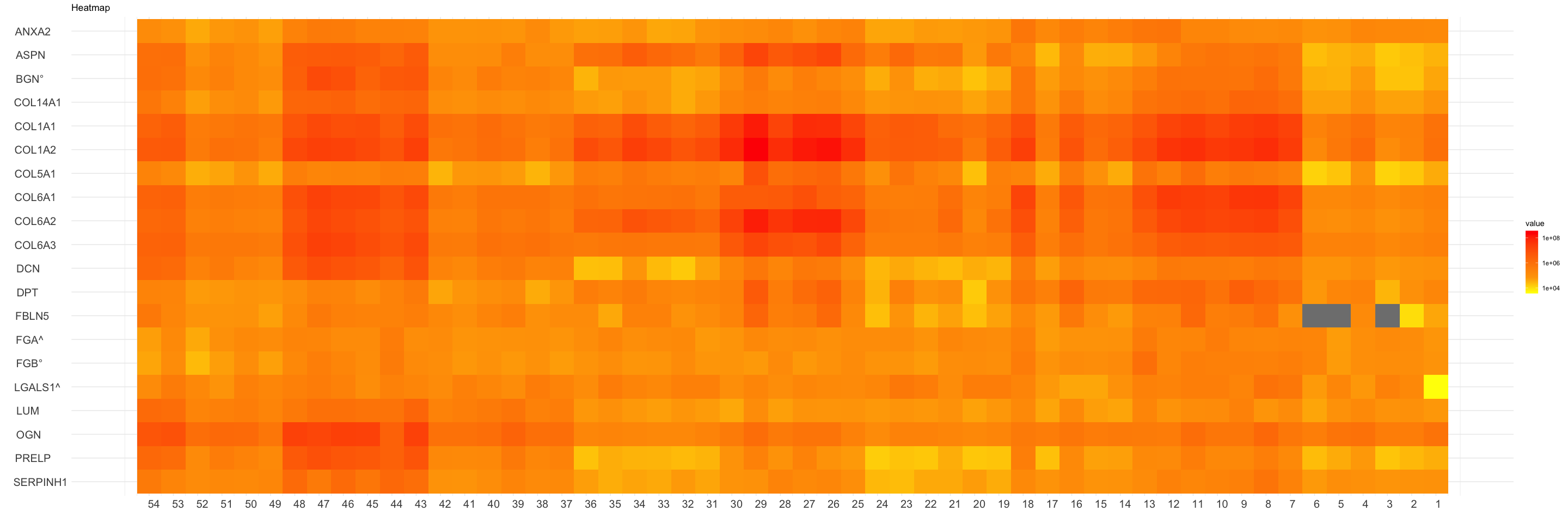

Supplement: Supplementary file 4 — Supplementary Material 5: Heatmap with all samples [file 13036_2025_602_MOESM4_ESM.docx]
