# Supplementary material for: Comparison of different decellularization protocols for porcine centrum tendineum diaphragmatis and diaphragmatic muscle – a base for effective recellularization
Source: J Biol Eng. 2026 Jan 7;20:16. doi: 10.1186/s13036-025-00602-z (PMC12836843; doi:10.1186/s13036-025-00602-z)

**Supplementary file 3 - list of matrisome proteins in subgroups**

A list of matrisome proteins and the subgroups in which they were preserved is shown.


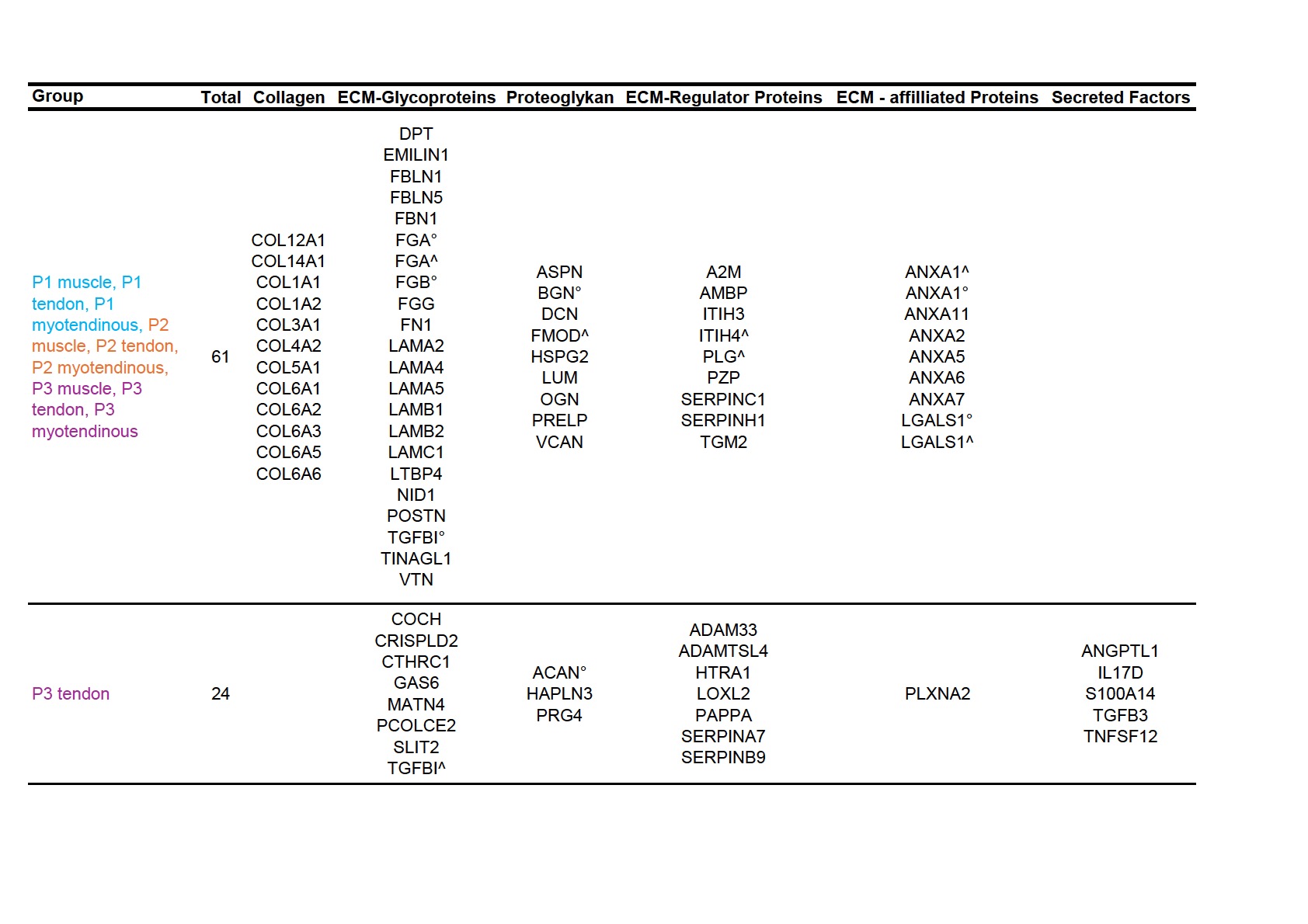

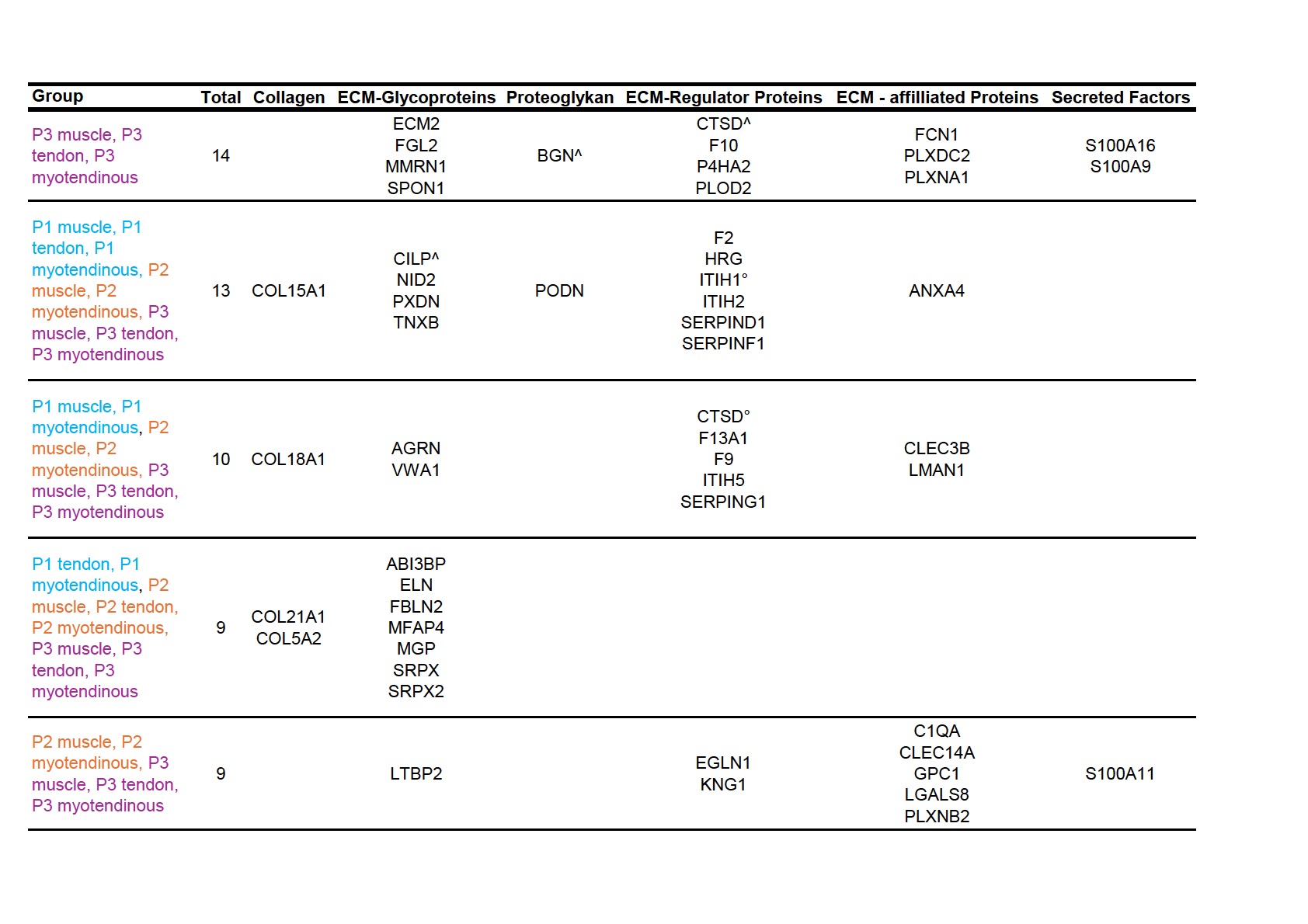

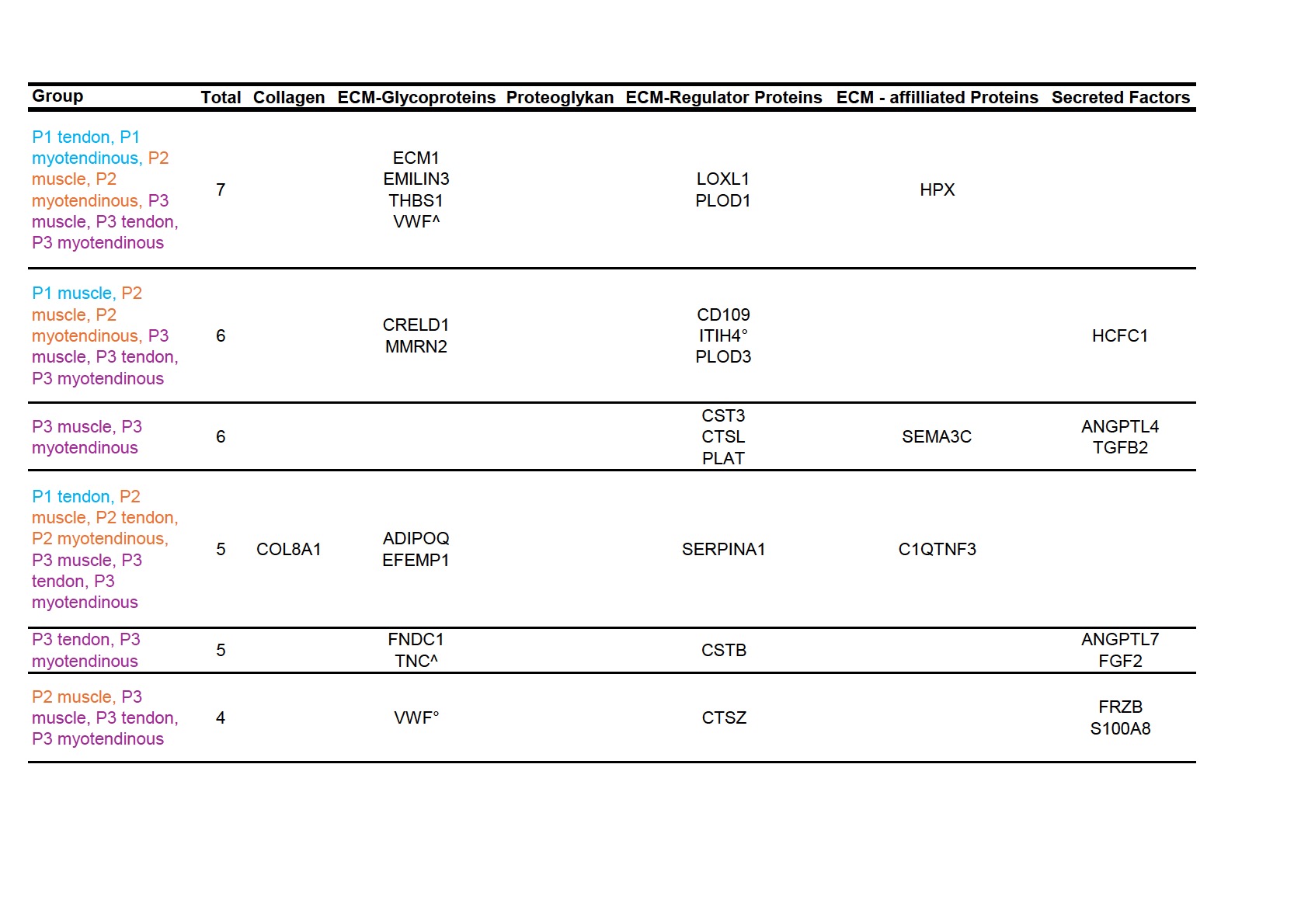

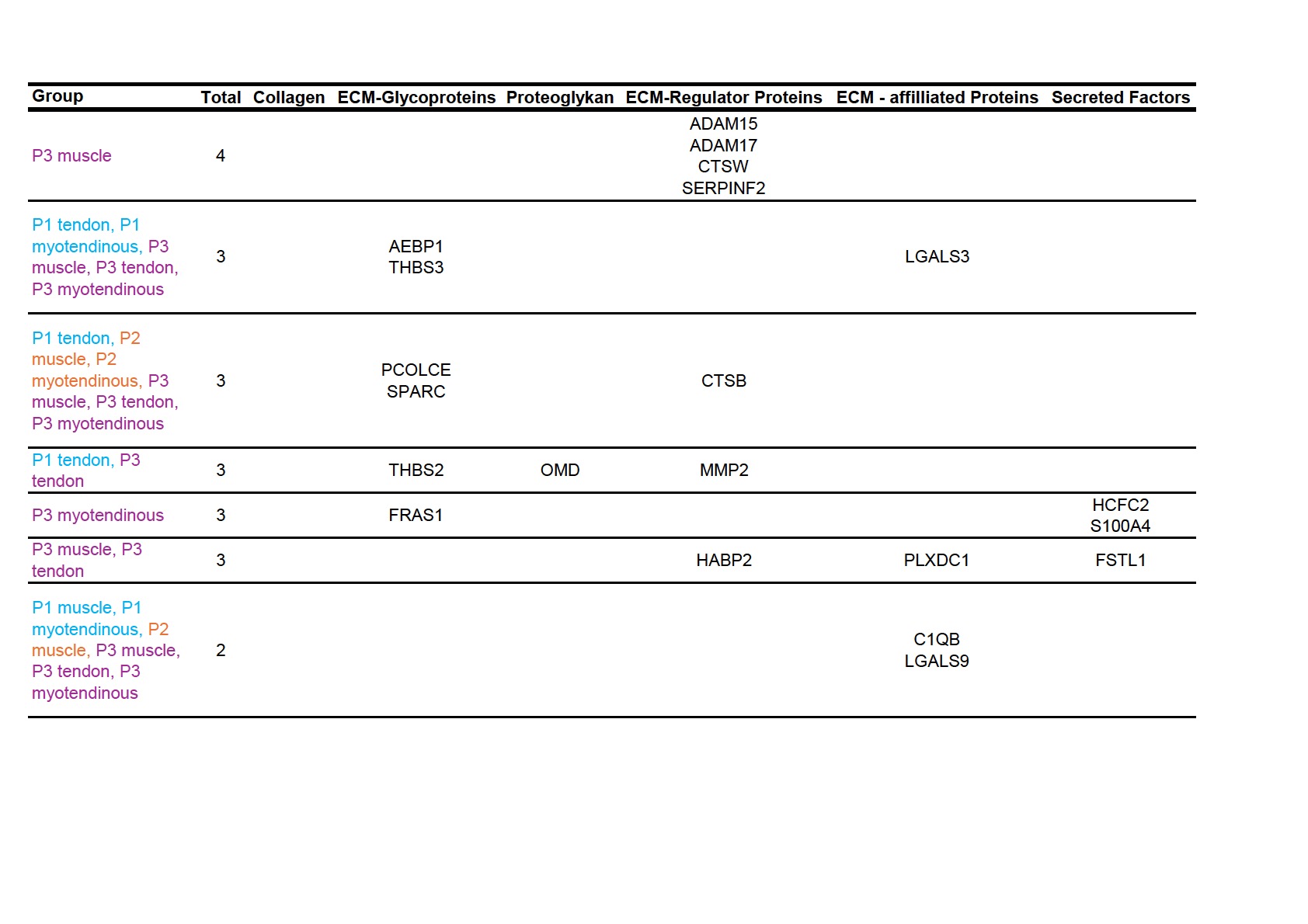

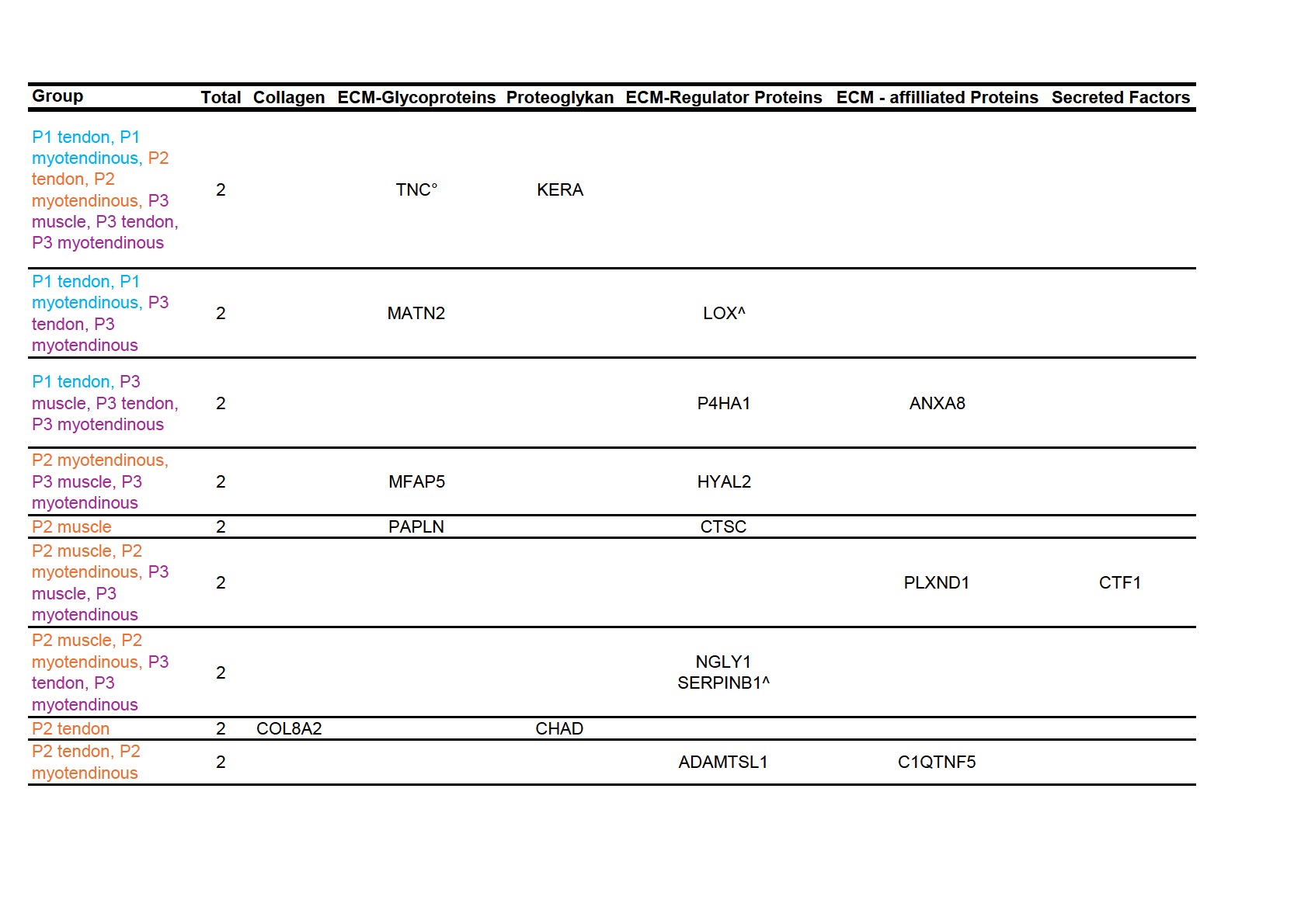

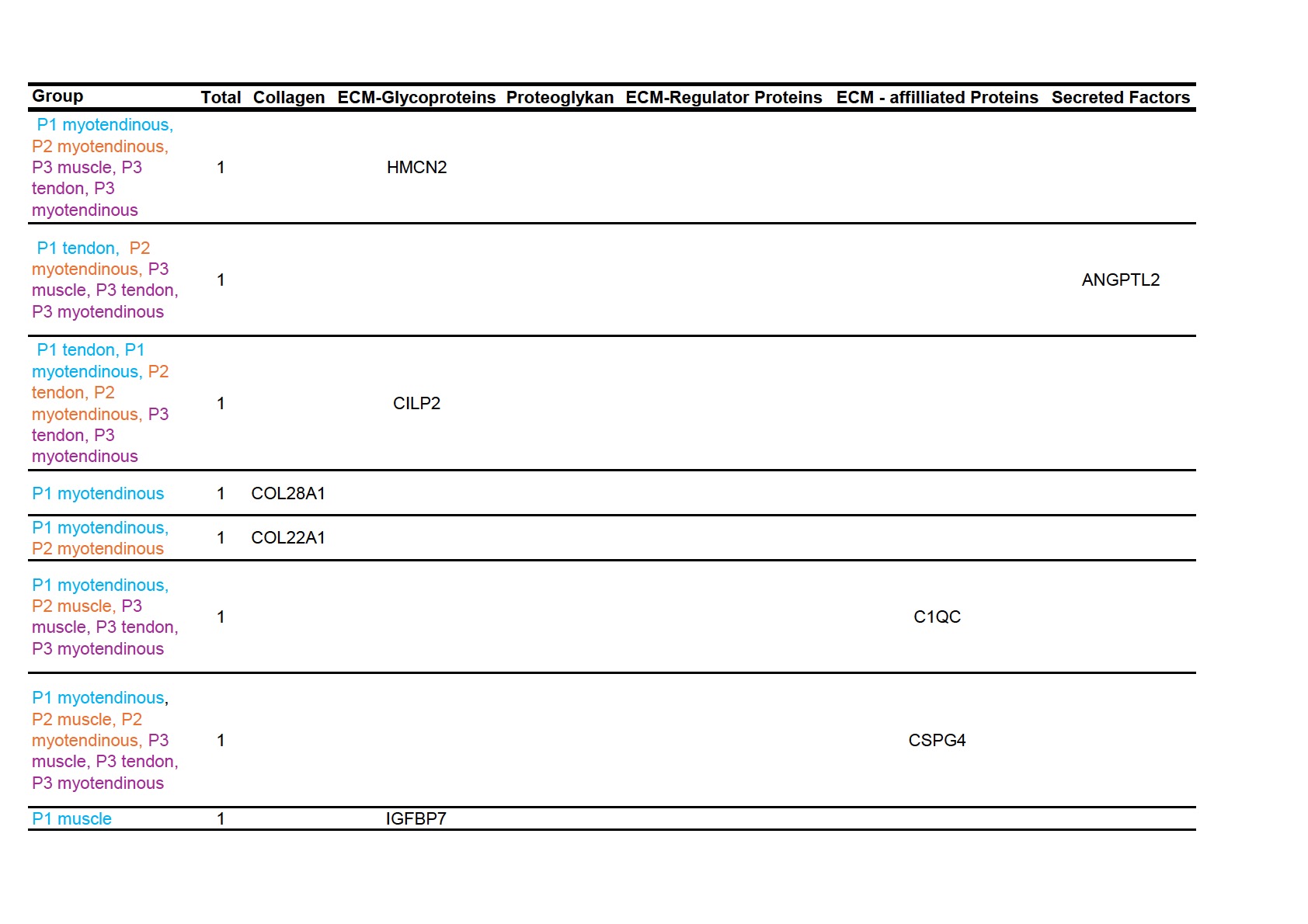

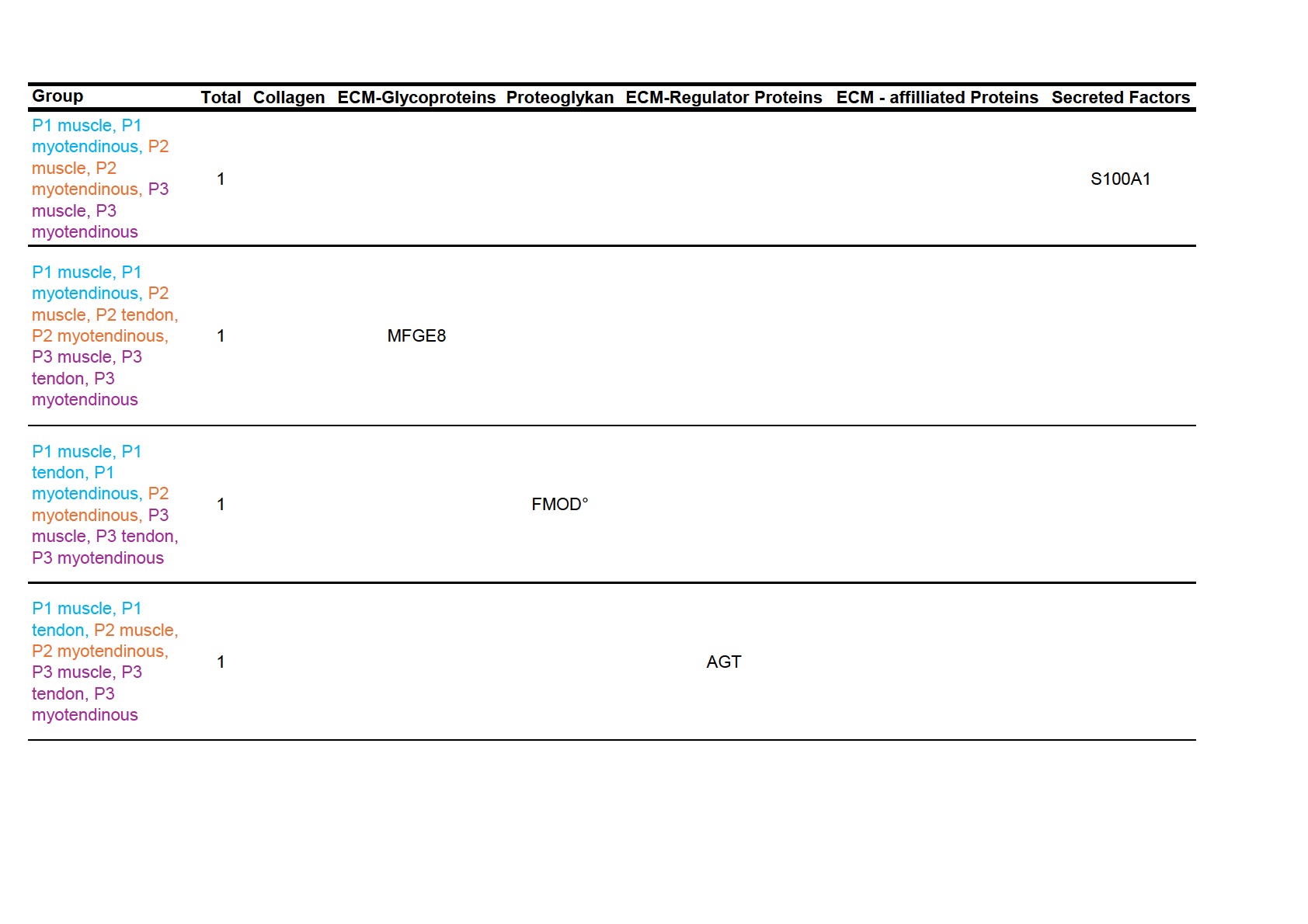

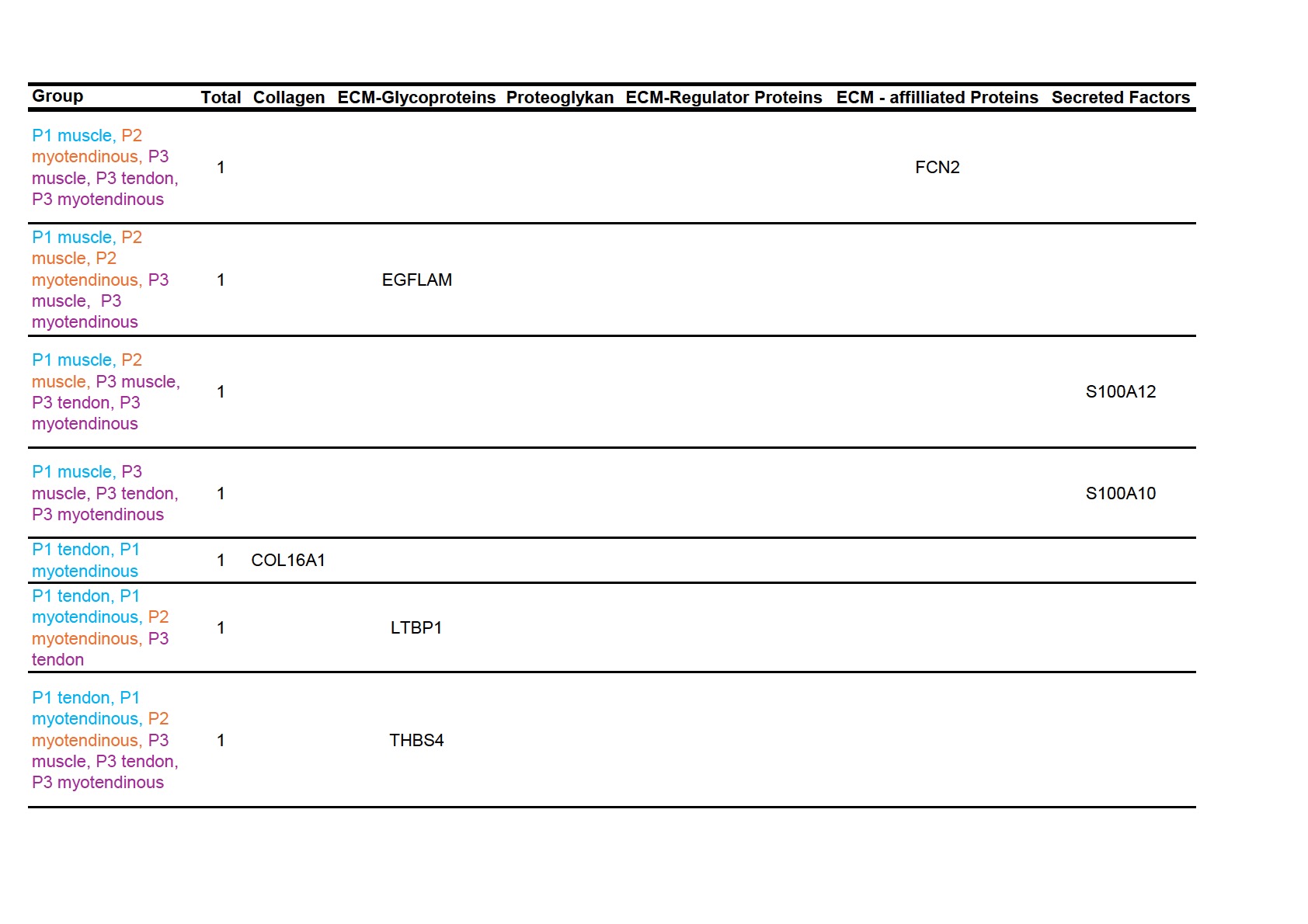

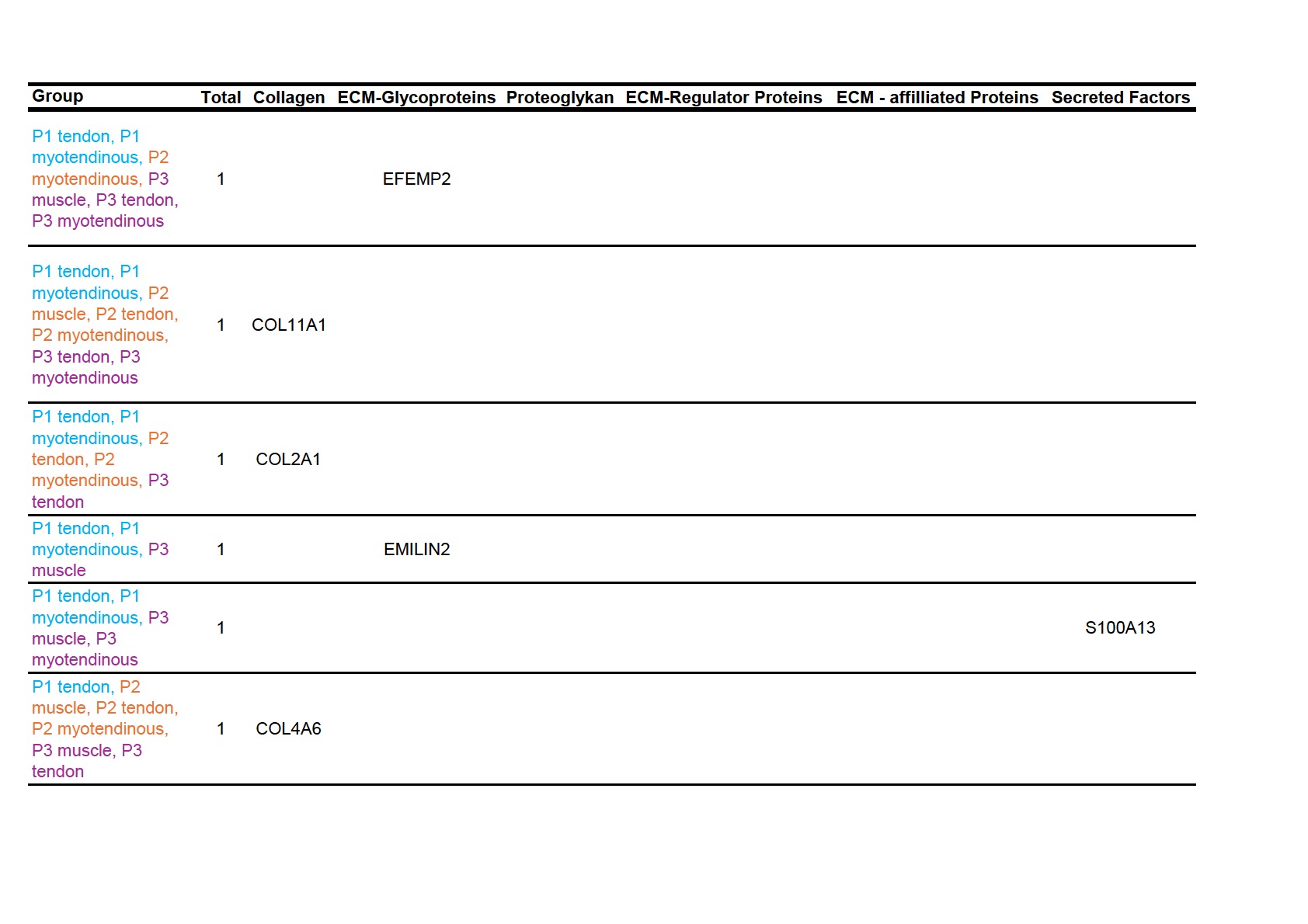

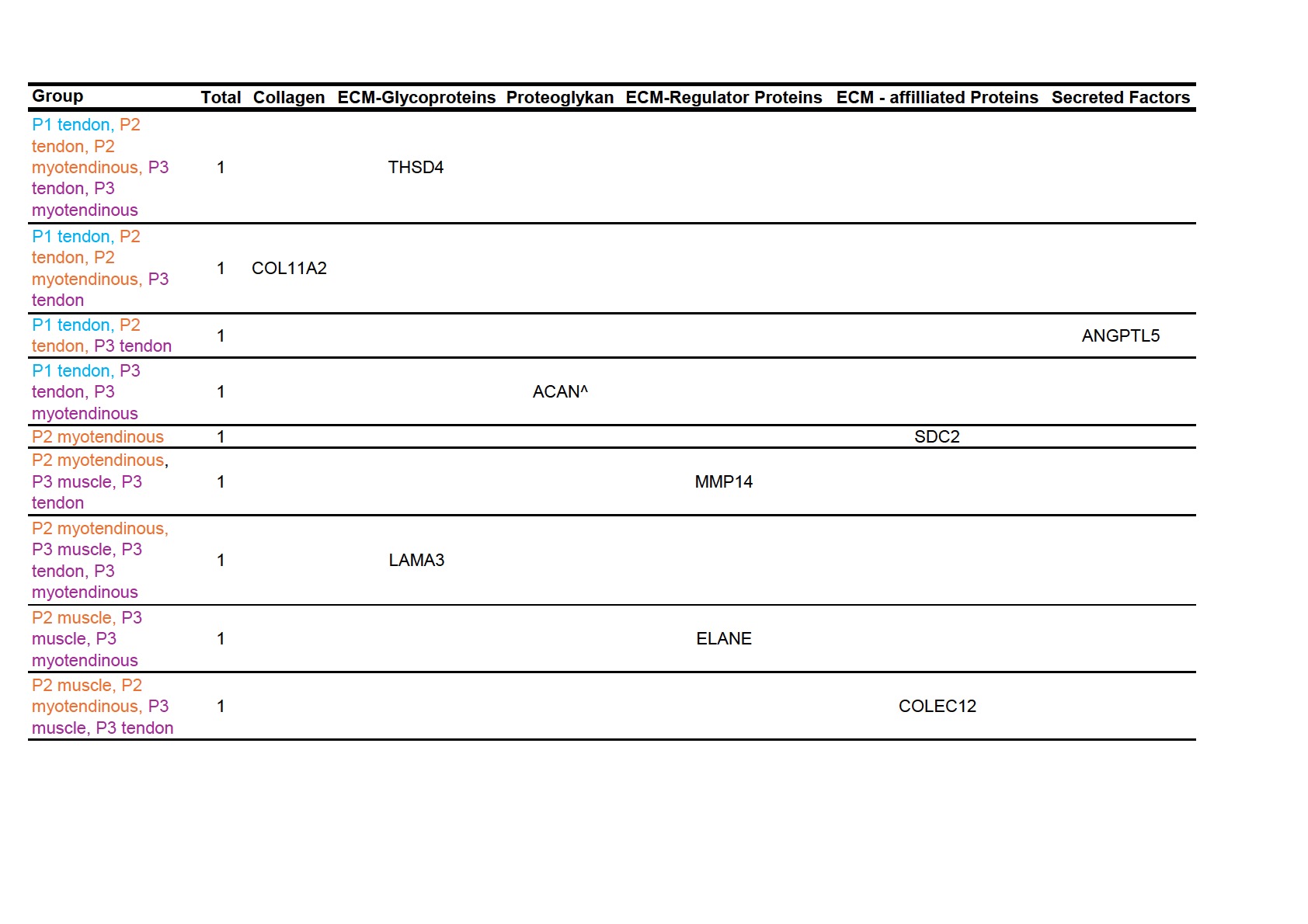

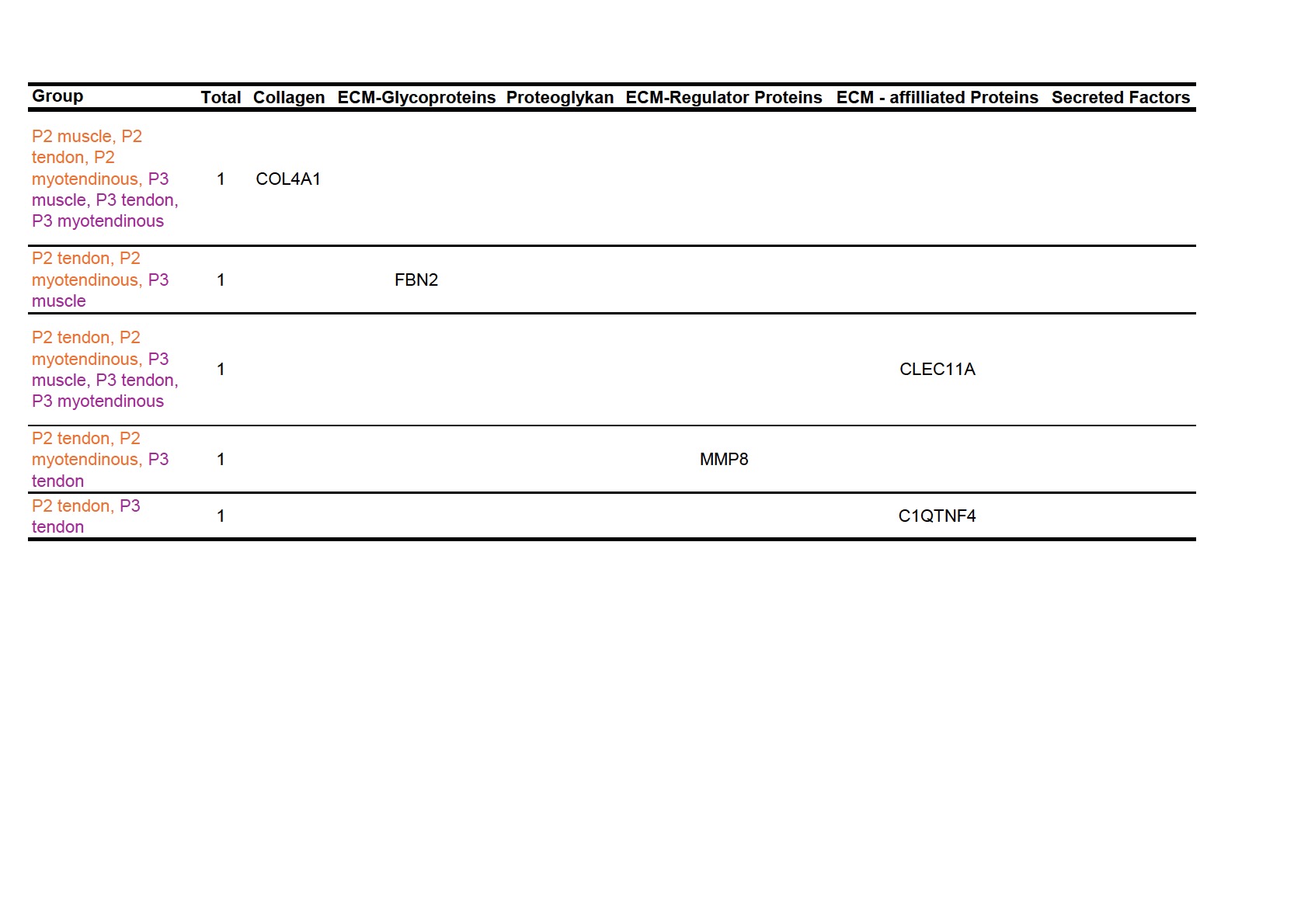

Supplement: Supplementary file 5 — Supplementary Material 6: List of matrisome proteins in subgroups [file 13036_2025_602_MOESM5_ESM.docx]
